# Natural history of facioscapulohumeral muscular dystrophy evaluated by multiparametric quantitative MRI: a prospective cohort study

**DOI:** 10.1007/s00415-025-13062-8

**Published:** 2025-04-02

**Authors:** M. Paoletti, M. Monforte, L. Barzaghi, G. Tasca, N. Bergsland, A. Faggioli, F. Solazzo, G. Manco, S. Bortolani, E. Torchia, B. Ravera, X. Deligianni, F. Santini, E. Ballante, S. Figini, T. Tartaglione, E. Ricci, A. Pichiecchio

**Affiliations:** 1 Advanced Imaging and Artificial Intelligence, Neuroradiology Department, IRCCS Mondino Foundation, Pavia, Italy; 2 Dipartimento di Neuroscienze, Organi di Senso e Torace, UOC di Neurologia, Fondazione Policlinico Universitario A. Gemelli IRCCS, Largo Agostino Gemelli, 8, 00168 Rome, Italy; 3 INFN, Group of Pavia, Pavia, Italy; 4 Department of Mathematics, University of Pavia, Pavia, Italy; 5 John Walton Muscular Dystrophy Research Centre, Newcastle University and Newcastle Hospitals NHS Foundation Trusts, Newcastle Upon Tyne, UK; 6 Department of Neurology, Jacobs School of Medicine and Biomedical Sciences, Buffalo Neuroimaging Analysis Center, University of Buffalo, the State University of New York, Buffalo, NY USA; 7 Università Cattolica del Sacro Cuore, Rome, Italy; 8 IRCCS, Fondazione Don Carlo Gnocchi ONLUS, Milan, Italy; 9 Department of Radiology, University Hospital Basel, Basel, Switzerland; 10 Department of Biomedical Engineering, Basel Muscle MRI, University of Basel, Basel, Switzerland; 11 Department of Political and Social Sciences, University of Pavia, Pavia, Italy; 12 BioData Science Center, IRCCS Mondino Foundation, Pavia, Italy; 13 Istituto Dermopatico Dell’Immacolata (IDI), IRCCS, Rome, Italy; 14 Department of Brain and Behavioural Sciences, University of Pavia, Pavia, Italy

**Keywords:** FSHD, Muscle, Fat fraction, WT2, Quantitative MRI, QMRI, Imaging

## Abstract

**Background:**

Facioscapulohumeral muscular dystrophy (FSHD) is a genetic disorder characterized by progressive skeletal muscle wasting. Longitudinal muscle magnetic resonance imaging (MRI) studies demonstrated that the risk of developing irreversible fatty replacement is higher in muscles showing edematous lesions. The quantification of this phenomenon is an understudied topic in FSHD and intramuscular water content can also represent a potential biomarker sensitive to the effect of investigational drugs. We applied a multiparametric quantitative muscle MRI protocol to assess disease progression quantifying fatty replacement and muscle edema over 2 years, using fat fraction (FF) and water-T2 (wT2) metrics.

**Methods:**

Thirty FSHD patients with at least one muscle showing signs of edema on conventional MRI were enrolled. FF and wT2 maps were assessed in 12 thigh and 6 leg muscles for each side, and a linear mixed model was employed to explore their variations over time. The measurements were acquired at baseline, 12, and 24 months. Quantitative MRI parameters were also correlated with clinical scales and functional assessments collected at baseline.

**Results:**

The average yearly increase in FF was 2 ± 0.6% at thigh level and 1.9 ± 0.7% at leg level. No significant longitudinal changes in wT2 were observed. Muscles with intermediate FF (15–30%) at baseline and those with baseline wT2 values above 41 ms showed the highest increase in fat replacement. Both FF and wT2 showed significant correlations with clinical scales and functional assessments.

**Conclusions:**

Our longitudinal study identified muscles and compartments more likely to show FF increase in FSHD subjects. Multiparametric quantitative MRI metrics should be incorporated into clinical trial frameworks to explore their potential in detecting early therapeutic effects.

**Supplementary Information:**

The online version contains supplementary material available at 10.1007/s00415-025-13062-8.

## Introduction

Facioscapulohumeral muscular dystrophy (FSHD) is an inherited neuromuscular disease characterized by slowly progressive and often asymmetric skeletal muscle wasting. Although it noticeably affects facial and scapular fixator muscles, lower limb, abdominal, and paraspinal muscles are also frequently involved [1].

Muscle magnetic resonance imaging (MRI) is an extremely valuable tool for the comprehensive investigation of muscle involvement in neuromuscular disorders [2–4], being able to provide accurate and reproducible information regarding the extent and distribution of intramuscular fat replacement and edema [5, 6]. In FSHD, a phase of “active” disease, characterized by a relative increase of intramuscular water content corresponding to fiber necrosis and inflammatory change on muscle pathology, directly linked to the molecular cascade of events causing muscle damage, can be identified through short tau inversion recovery (STIR) sequences on conventional muscle MRI [7–10].

The presence of STIR-positive muscles (i.e., muscles with areas of hyperintense signal on STIR) has been linked to an increased risk of radiological worsening and progressive fatty replacement [11–13]. Nevertheless, the aforementioned alterations cannot be precisely quantified through the application of STIR sequences, which are inherently qualitative in nature.

The development of advanced muscle MRI techniques has enabled greater precision in the characterization of pathological changes in muscle tissue, including the quantification of the degree of fat replacement and intramuscular edema, as indicated by metrics such as fat fraction (FF) and water-T2 (wT2) [5, 6]. Fat fraction has been extensively used in the muscle imaging field as a powerful indicator of chronic and irreversible muscle damage, whereas the use of wT2 to capture changes related to the acute phase of disease worsening has been so far more limited and difficult to interpret.

The average annualized increase in intramuscular fat fraction broadly varies in the different studies dealing with FSHD, from 1.0 to 6.7% [14–17]. A recent study in a large cohort of patients showed an even lower rate (global median change of 2% over 5 years in a compound score of mean fat fraction weighted for cross sectional area) [18]. Conversely, quantification of the water-sensitive metrics has not been studied as extensively in FSHD [19]. Development and exploitation of such metrics are particularly relevant as the effect of some of the investigational products under development may be only reflected by water mobility changes in an early phase. As quantitative MRI biomarkers are currently being employed as exploratory endpoints in ongoing therapeutic clinical trials for this disease [20, 21], there is a need for additional data to establish natural history values. Moreover, if radiological disease progression or activity are among the targets of an investigational drug, the ideal approach for detecting meaningful differences in a clinical trial is to prioritize enrolment of patients exhibiting a rapidly worsening trend in their natural history.

Following this reasoning, we decided to select a cohort of FSHD patients with “active” disease (i.e., subjects with at least one muscle hyperintense on STIR on a qualitative baseline MRI), theoretically fast-progressing according to actual knowledge, and to follow them by a multiparametric quantitative muscle MRI protocol assessing the lower limbs over 2 years. Our aims were (1) to evaluate the longitudinal changes in FF and wT2 in this specific cohort of FSHD subjects in individual muscles, and (2) to assess the predictivity of baseline quantitative MRI metrics with regards to the evolution of the disease.

## Materials and methods

### Study design and subjects

This longitudinal study was performed from 2018 to 2022. Adult subjects (> 18 years old) followed at the Fondazione Policlinico Universitario A. Gemelli hospital and affected by genetically confirmed FSHD type 1 were eligible if presenting at least one STIR positive muscle on standard muscle MRI examination (axial 2D T1-weighted and STIR imaging sequences), performed before enrollment. Two authors (MM and ER) visualized the scans and qualitatively evaluated the presence/absence of STIR positive muscles. At baseline clinical visits, we collected the following: demographics, EcoRI fragment length, Clinical Severity Score (CSS) [22], 6-min walking test (6MWT) and muscle strength using a hand-held dynamometry (Hoggan microFET2^®^, movements: knee extension and flexion). A baseline (t0) quantitative MRI examination was then performed and repeated after 12 (t12) and 24 months (t24). The study was conducted in accordance with the Declaration of Helsinki and its later amendments and was approved by the local institutional review board (Prot. 7451/18 ID:1952). All participants signed written informed consent prior to participating in the study.

### MRI acquisition

Quantitative muscle MRIs were acquired at IRCCS Mondino Foundation in Pavia, Italy. At each time point all subjects underwent a quantitative muscle MRI protocol (3 T Skyra scanner, Siemens Healthcare, Erlangen, Germany), with two 18-channel phased-array coils applied respectively on thighs and lower legs. The subjects were lying in the supine position with the leg stretched out.

The protocol included an axial 6-point 3D Dixon gradient echo (GRE) sequence (matrix size = 432 × 396; TR = 35 ms; TE = 1.7–9.2 ms; resolution = 1.0 × 1.0 × 5 mm3; flip angle = 7°; slice oversampling = 38.5%; 52 slices for the thigh, 26 for the leg; scan time = 15 min for the thigh and 10 for the leg) and an axial multi-echo turbo spin echo (TSE) T2 sequence (TE/TR = [10.9–185.3] ms /4100.0 ms, 17 echo times; resolution = 1.2 × 1.2 × 10.0 mm3; slice gap = 30 mm; no fat suppression; 7 slices for the thigh, 5 for the leg; scan time = 5 min). Vitamin E capsules were placed upon the skin of the patient as positioning markers to allow cross sectional intra-cohort reproducibility and longitudinal intrasubject reproducibility. For intra-cohort reproducibility, a vitamin E capsule was positioned on the patient's skin at one-third of the total distance between the upper edge of the patella and the ipsilateral anterior superior iliac spine. For longitudinal within-subject reproducibility, the precise capsule placement (measured in cm) from the initial examination was recorded and replicated in all subsequent scans.

### Image processing

**Fig. 1:**
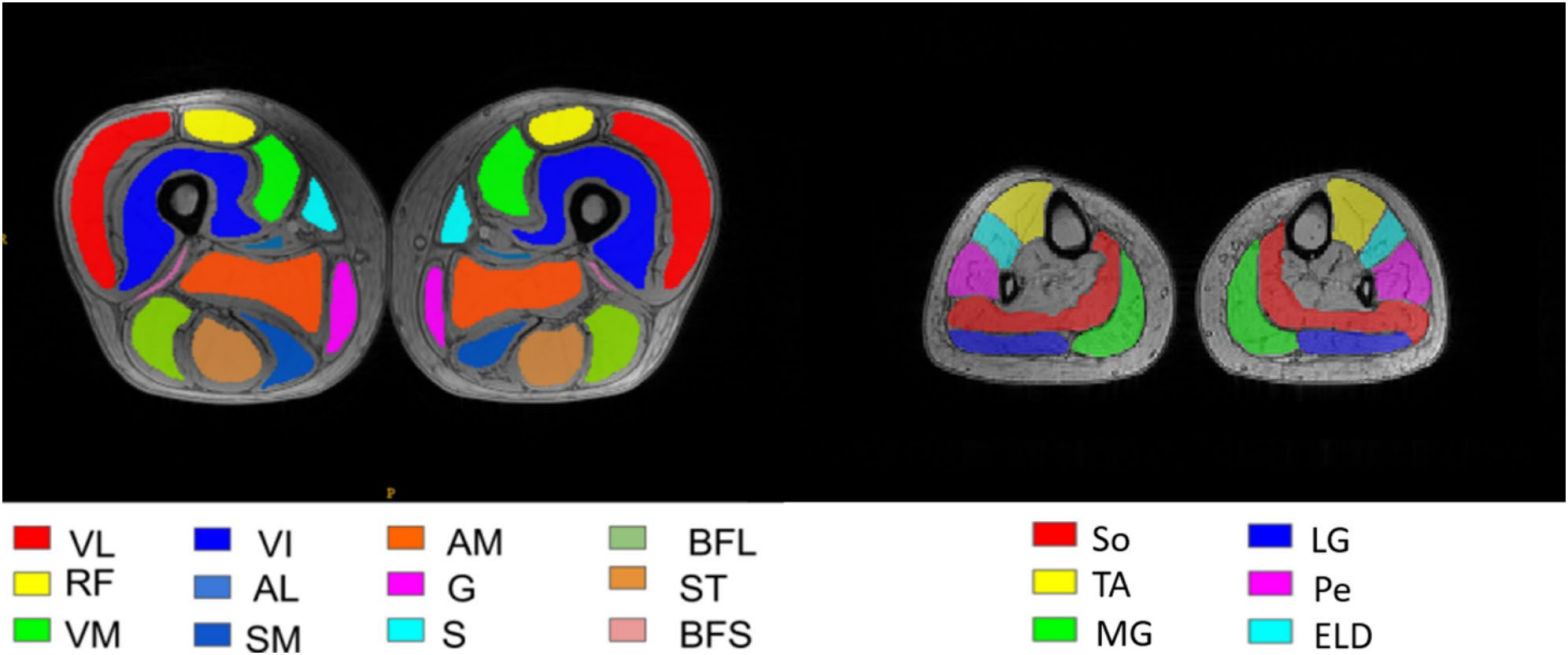
Example of muscle segmentation for thigh and leg superimposed on an axial Dixon image. The color code is reported below for the following thigh muscles: VL, vastus lateralis; RF, rectus femoris; VM, vastus medialis; VI, vastus intermedius; AL, adductor longus; SM, semimembranosus; AM, adductor magnus; G, gracilis; S, sartorius; BFL, long head of biceps femoris; ST, semitendinosus; BFS, short head of biceps femoris. The anterior compartment of the thigh includes VL, VM, VI, and RF; medial compartment of the thigh includes S, G, AM, and AL; the posterior compartment of the thigh includes SM, ST, BFL, and BFS. Leg muscles: So, soleus; TA, tibialis anterior; MG, medial gastrocnemius; LG, lateral gastrocnemius, Pe, peroneus; ELD, extensor digitorum longus. The anterior-lateral compartment of the leg includes TA, ELD, and PE; the posterior compartment of the leg includes S, GM, and GL

Muscle segmentation was performed on the T2 TSE sequence (first echo of the sequence) using the open-source Dafne platform (https://dafne.network/, version 1.3-alpha2) [23, 24]. The Dafne software has been further trained with in-house data to boost the segmentation performances on the current FSHD dataset. We studied 12 different muscles at the level of the thigh and 6 different muscles at the level of the calf, bilaterally (Fig. 1). At thigh level, the studied muscles were vastus intermedius (VI), vastus lateralis (VL), vastus medialis (VM), rectus femoris (RF), sartorius (Sa), gracilis (G), adductor magnus (AM), adductor longus (AL), semitendinosus (ST), semimembranosus (SM), biceps femoris long head (BFL), biceps femoris short head (BFS). At leg level we studied the following muscles: tibialis anterior (TA), extensor longus digitorum (ELD), personal muscles (Pe), soleus (So), gastrocnemius medialis (MG), gastrocnemius lateralis (LG).

All generated regions of interest (ROIs) by Dafne were visually reviewed and corrected (if necessary) by a single expert operator with > 5 years of experience in muscle segmentation, using the open-source software ITKSNAP [25]. The middle slices of the TSE T2 sequence were considered for segmentation for the thigh (*n* = 5/7) and leg (*n* = 3/5); the first and last slices were excluded. All ROIs were subsequently registered to Dixon images, doubling the number of segmented slices. The expert operator corrected (if necessary) the registered ROIs on the Dixon slices, again using ITKSNAP [25].

The average fat fraction (FF) was calculated for the different ROIs from the 6-point Dixon images applying the open-source algorithm FattyRiot [26]. The algorithm models overlapping complex signals from fat and water at different echo times in a multi-echo GRE acquisition. FattyRiot models the multi-peak fat spectrum and the water peak, along with the effects of field inhomogeneities and T2* decay, and ultimately provides separate fat and water images. The FF image is the ratio between the fat signal and the sum of fat and water signals in each voxel. In addition, water T2 (wT2) was then calculated using an extended-phase-graph fitting approach with the multi-echo T2 spin-echo data [27, 28]. The method accounts for complex spin dynamics, including signal decay, dephasing, and stimulated echoes and ultimately fits the water signal component, resulting in a wT2 map.

For the analysis, the ROIs from different muscles were grouped together to obtain a combined value for the lower limbs of each patient, values for the whole thigh and whole leg, and values for different compartments (anterior thigh, medial thigh, posterior thigh, anterior-lateral leg, and posterior leg). In more detail, the average FF and wT2 within the whole thigh and whole leg were then obtained using the combined mask of the individual muscle ROIs.

**Fig. 2:**
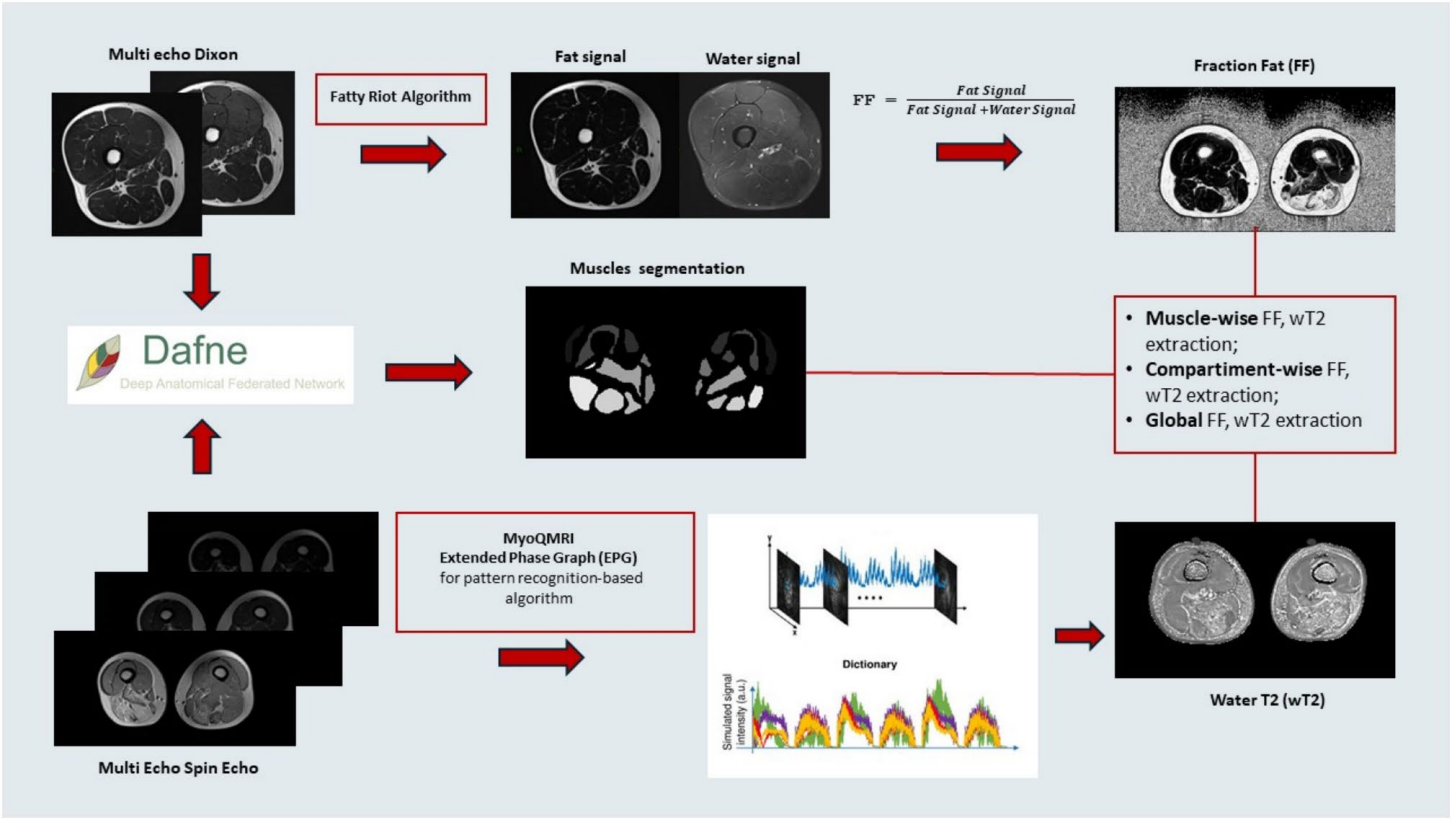
Pictorial description of the workflow adopted for extracting FF and wT2 parameters maps from thigh MRI images. Multi echo Dixon and Spin Echo images were processed using Fatty Riot and MyoQMRI to generate fat fraction (upper section) and water T2 (lower section) parameter maps. Muscle segmentation masks (middle section) were created using both the Multiecho Dixon and Spin Echo images by Dafne segmentation software, which were subsequently overlaid on the parameter maps to extract muscle-wise, compartment-wise, and global FF and wT2 values

The whole process of post-processing of quantitative maps is summarized in Fig. 2.

### Statistics

Statistical analyses were performed with Python v.3.9.0 using Statsmodels v.0.13.2 and SciPy v.1.9.0 libraries.

We used linear mixed models (LMM) to investigate FF and wT2 variations over time. As random effects, we included subject-wise dependency both on FF and wT2 baseline values (random intercepts) and longitudinal variations (random slope). We used baseline, 12 and 24 months FF and wT2 as input to LMM. The annualized delta-FF and delta-wT2 were thus obtained for each muscle, the different compartments and the whole-thigh and whole-leg.

We explored the influence of baseline FF and wT2 values on the annualized delta-FF. We clustered our dataset into tertiles based on baseline FF (< 15%, 15% < FF < 30%, > 30%) to evaluate the evolution of fatty replacement over time for each group, using the same LMM approach. Literature thresholds [29] were used for each tertile. We further used a more granular data-driven approach, evaluating the annualized delta-FF in relation to the baseline FF divided into 10 bins (from 0–10% to 90–100%).

Similarly, we dichotomized our dataset in normal or elevated wT2, applying a threshold of 41 ms, a value derived from a cohort of healthy subjects who previously underwent a quantitative muscle MRI protocol on the same scanner and also consistently with the values used in the literature [19, 30].

For both FF and wT2-based subgroups, Kruskal–Wallis and post-hoc correction were used to assess inter-groups differences in fat content progression over time.

For correlation between FF, wT2 and clinical outcomes we used Pearson correlation test. For all the analyses *p*-value < 0.05 were set as the significant threshold.

## Results

### Participants

Thirty FSHD subjects (18 males and 12 females, aged 45 ± 9 years) were enrolled. Follow up was acquired after 14.1 months ± 54 days for the t12 timepoint and after 26.2 months ± 80 days for the t24 one. The small deviation from the planned 12- and 24-months follow-up was due to the COVID-19 pandemic and related restrictions that were applied in Italy from March 2020. All 30 subjects underwent muscle MRI examination at the scheduled three time-points. Baseline clinical characteristics and evaluations are summarized in Table 1.

**Table 1:** Demographics and clinical data

	Mean ± SD or Median [IQR]	Range
Age (y)	45 ± 9	19–60
EcoRI fragment lenght (kb)	25 ± 6	10–38
CSS	3 [1]	1–4.5
6MWT (m)	436 ± 136	65–670
STIR-positive muscles (n per patient)	10.3 ± 5.6	1–23
Dynamometry (kgf)		
Knee extensors right	27.9 ± 14.1	7–61.6
Knee extensors left	29.2 ± 13.5	5.9–59.1
Knee flexors right	20.1 ± 11.9	0.8–40.1
Knee flexors left	18.9 ± 10.5	0.8–40.5

*SD* standard deviation, *IQR* interquartile range, *y* years, *kb* kilobase, *CSS* clinical severity score, *6MWT* six-minutes walking test, *m* meters, *STIR* short-tau inversion recovery, *n* number, *kgf* kilogram-force

### Fat-fraction

For every timepoint, a total number of 1080 muscles were analyzed. The average fat-fraction at baseline was 22.4% ± 2.7% for thigh muscles and 22.1% ± 3.0% for leg muscles. The muscle with the highest fat-fraction was SM (53.1% ± 3.4%), followed by MG (42.3% ± 3.7%) and AL (40.2% ± 5.8%). The least fatty replaced muscles were VL (10.6% ± 1.2%), Pe (11.8% ± 1.6%) and VI (14.5% ± 2.7%). The posterior thigh had the highest fat-fraction compartment-wise (36.2% ± 3.0%).

Over the 24-month study period, the muscles that showed a statistically significant increase in their fat-fraction were RF (annualized delta-FF + 5.1% ± 1.5%, *p* < 0.001), VI (+ 2.8% ± 0.8%, *p* < 0.001), ST (+ 2.7% ± 1.2%, *p* < 0.05) and So (+ 1.5% ± 0.7%, *p* < 0.05). All compartments showed a significant yearly increase of FF, with the exception of the medial thigh one (*p* = 0.07). At the whole thigh level, the annualized delta FF was + 2.0% ± 0.6% (*p* < 0.001), while at the whole leg level was + 1.9% ± 0.7% (*p* < 0.05) (Table 2).

**Table 2:** Longitudinal evolution of fat fraction (FF) and water T2 (wT2) at whole-thigh and whole-leg level and at compartment- and single-muscle level

District	FF baseline (%)	DeltaFF (% yearly)	wT2 baseline (ms)	Delta wT2 (ms yearly)
Whole-thigh	22.4% ± 2.7%	+ 2.0% ± 0.6% ***	42.9 ± 0.5 ms	+ 0.13 ± 0.28 ms
Whole-leg	22.1% ± 3.0%	+ 1.9% ± 0.7% *	43.4 ± 0.6 ms	−0.16 ± 0.36 ms
Anterior thigh	20.0% ± 2.4%	+ 3.0% ± 0.8% ***	42.1 ± 0.7 ms	+ 0.30 ± 0.23 ms
VI	14.5% ± 2.7%	+ 2.8% ± 0.8% ***	42.6 ± 0.7 ms	+ 0.33 ± 0.26 ms
VL	10.6% ± 1.2%	+ 1.1% ± 0.7%	41.8 ± 0.9 ms	+ 0.14 ± 0.73 ms
VM	20.0% ± 3.8%	+ 1.6% ± 1.0%	43.3 ± 0.8 ms	+ 0.26 ± 0.22 ms
RF	32.7% ± 5.2%	+ 5.1% ± 1.5% ***	39.4 ± 0.8 ms	+ 0.35 ± 0.40 ms
Medial thigh	30.2% ± 3.6%	+ 1.9% ± 1.1%	43.6 ± 0.5 ms	−0.20 ± 0.22 ms
Sa	20.3% ± 2.9%	+ 1.6% ± 1.4%	42.9 ± 0.6 ms	−0.29 ± 0.20 ms
G	25.7% ± 4.5%	+ 1.5% ± 1.2%	41.2 ± 0.6 ms	−0.21 ± 0.25 ms
AM	33.4% ± 4.7%	+ 1.1% ± 1.4%	45.3 ± 0.6 ms	+ 0.21 ± 0.37 ms
AL	40.2% ± 5.8%	+ 2.4% ± 1.8%	45.1 ± 0.8 ms	−0.53 ± 0.58 ms
Posterior thigh	36.2% ± 3.0%	+ 1.7% ± 0.8% *	43.1 ± 0.5 ms	−0.225 ± 0.41 ms
ST	35.4% ± 5.4%	+ 2.7% ± 1.2% *	41.3 ± 0.5 ms	−0.17 ± 0.52 ms
SM	53.1% ± 3.4%	+ 1.3% ± 1.3%	44.1 ± 0.6 ms	−0.29 ± 0.47 ms
BFL	30.1% ± 4.7%	+ 2.4% ± 1.3%	47.0 ± 2.0 ms	−2.20 ± 1.23 ms
BFS	27.4% ± 4.1%	−0.0% ± 1.0%	43.1 ± 0.5 ms	+ 0.08 ± 0.33 ms
Anterior-lateral leg	24.7% ± 2.4%	+ 1.6% ± 0.7% *	42.5 ± 0.6 ms	−0.39 ± 0.73 ms
TA	34.4% ± 3.3%	+ 1.0% ± 2.0%	43.5 ± 0.8 ms	−0.21 ± 0.75 ms
ELD	30.4% ± 4.5%	+ 0.3% ± 1.1%	42.8 ± 0.7 ms	−0.45 ± 0.79 ms
Pe	11.8% ± 1.6%	+ 1.7% ± 1.2%	40.9 ± 0.4 ms	−0.51 ± 0.67 ms
Posterior leg	25.5% ± 2.4%	+ 1.4% ± 0.7% *	43.4 ± 0.6 ms	−0.09 ± 0.26 ms
So	16.1% ± 3.0%	+ 1.5% ± 0.7% *	44.4 ± 0.7 ms	−0.30 ± 0.29 ms
MG	42.3% ± 3.7%	+ 1.6% ± 1.9%	44.2 ± 0.8 ms	−0.10 ± 0.36 ms
LG	18.2% ± 3.8%	+ 1.0% ± 1.0%	41.2 ± 0.6 ms	−0.02 ± 0.52 ms

Data are presented as yearly increase as assessed through the Linear Mixed Model (LMM) over the time frame of 24 months. Baseline FF (%) and wT2 (ms) are also presented, with standard deviations. P values are reported as follows: *(< 0.05), **(< 0.005), ***(< 0.001). Statistically significant differences are reported in bold

### Water-T2

The average baseline wT2 value of all individual muscles was 43.17 ± 5.46 ms. The average wT2 value of STIR-positive muscles was 45.93 ± 6.9 ms and resulted statistically significantly higher than the value of STIR-negative ones (41.97 ± 4.22 ms, *p* value < 0.001). BFL resulted in the muscle with the highest baseline wT2 value (47.0 ± 2.0 ms), followed by AM (45.3 ± 0.6 ms) and AL (45.1 ± 0.8 ms).

The longitudinal evaluation of wT2 values did not show any significant change at single muscle, compartment and whole thigh or leg level (Table 2).

### Baseline FF and wT2 as predictors of delta-FF

The muscles that showed the highest statistically significant change in the annualized delta-FF were the ones with baseline fat-fraction between 15 and 30% (Table 3). In particular, the gracilis muscle (G) from this subgroup displayed the highest progression among all (annualized delta-FF + 20.5%, *p* < 0.005). Several muscles with a baseline FF > 30% also showed significant changes (for example VI, + 6.3%, *p* < 0.001). The subgroup of muscles with baseline FF between 15 and 30% showed also statistically significant annualized delta-FF increase in almost all studied compartments and both at whole thigh and leg level (Table 3). The detailed analysis of the yearly FF change in relation to the baseline FF value, divided in percentiles, showed that in the > 30% bin coexist muscles that still show remarkable FF changes (for example in SM, AM or BFL and BFS) with non-progressive muscles due to very high fatty replacement at baseline (Supplementary Figures).

**Table 3:** Yearly FF variation for subgroup 1, 2 and 3 defined according to the baseline FF (FF_b_)

Region	Group1 (FF_b_ < 15%)	Group2 (15% < FF_b_ < 30%)	Group3 (FF_b_ > 30%)
Whole thigh	+ 1.07% (14)	+ 2.73% * (9)	+ 2.96% * (8)
Whole leg	+ 0.58% * (11)	+ 3.27% * (13)	+ 1.89% (6)
Anterior thigh	+ 0.92% (12)	+ 2.63% * (8)	+ 5.81% *** (6)
VI	+ 0.93% (18)	+ 6.5% ** (5)	+ 6.3% *** (5)
VL	+ 1.42% (16)	+ 0.78% (8)	∄
VM	+ 0.61% (17)	+ 11.5% ** (2)	+ 4.1% ** (8)
RF	+ 6.92% (10)	+ 9.4% ** (5)	+ 2.2% * (14)
Medial thigh	+ 2.03% (8)	+ 3.37% * (18)	+ 0.37% (10)
Sa	+ 1.64% (7)	+ 2.74% (13)	+ 0.31% (5)
G	0.00% (16)	+ 20.5% ** (2)	+ 0.76% (9)
AM	+ 0.72% (4)	+ 2.5% * (11)	+ 1.02% (14)
AL	+ 8.73% * (6)	+ 7.9% (6)	− 0.70% (12)
Posterior thigh	+ 1.14% ** (5)	+ 2.92% ** (10)	+ 1.63% (10)
SM	+ 1.92% (3)	+ 2.3% ** (3)	+ 1.10% (23)
ST	+ 2.51% (10)	+ 7.2% *** (6)	+ 0.07% (13)
BFL	+ 1.26% * (12)	+ 5.72% (11)	+ 1.81% (7)
BFS	+ 1.42% (8)	− 1.70% (11)	− 1.17% (11)
Anterior-lateral leg	+ 0.64% * (9)	+ 3.22% *** (6)	+ 1.31% (10)
TA	+ 0.9% *** (10)	+ 2.30% (7)	+ 0.20% (13)
ELD	+ 1.0% * (13)	+ 3.70% (5)	− 0.20% (11)
Pe	+ 1.6% (15)	+ 6.00% (12)	∄
Posterior leg	0.00% (9)	+ 2.12% (7)	+ 2.16% (10)
So	+ 1.65% (12)	+ 5.2% *** (11)	− 0.41% (6)
MG	+ 0.9% (5)	− 0.23% (6)	+ 2.21% * (16)
LG	+ 2.34% (12)	0.00% (12)	+ 0.32% (4)

*P* value: * (< 0.05), **(< 0.005), ***(< 0.001), ∄: no data available. Statistically significant differences are reported in bold. In brackets the number of muscles in each group

With respect to baseline wT2 values, a significant change in annualized delta-FF was documented for 5 out of 18 muscles with baseline wT2 values > 41 ms, while only in two cases this occurred for baseline wT2 values < 41 ms (Table 4). It is worth noting that RF, the muscle with the highest increase in FF among all, maintained the significance of FF change in both groups stratified based on baseline wT2 values. No significant change in FF was found for all the studied compartments and at whole thigh and leg level for the subgroup of muscles with baseline wT2 values < 41 ms, while for the other subgroup (baseline wT2 values > 41 ms) the difference was significant for all compartments (except the medial thigh one) and at whole thigh and leg level (Table 4).

**Table 4:** Yearly FF variation over time for subgroup A and B defined according to the baseline wT2 (wT2_b_) value

Region	Group A (wT2_b_ ≤ 41 ms)	Group B (wT2_b_ > 41 ms)
Whole thigh	+ 0.45% (6)	+ 2.46% * (24)
Whole leg	0.00% (3)	+ 2.24% * (26)
Anterior thigh	+ 0.56% (10)	+ 4.24% *** (20)
VI	+ 0.19% (4)	+ 4.12% *** (22)
VL	+ 1.14% (6)	+ 1.48% (23)
VM	+ 0.23% (7)	+ 2.57% *** (21)
RF	+ 5.12% * (11)	+ 5.01% ** (19)
Medial thigh	+ 1.51% (6)	+ 2.10% (23)
Sa	− 0.03% (4)	+ 2.48% (22)
G	− 0.01% (5)	+ 2.91% (19)
AM	+ 1.48% * (8)	+ 1.12% (21)
AL	+ 0.72% (4)	+ 1.64% (25)
Posterior thigh	+ 0.93% (5)	+ 2.92% *** (23)
SM	+ 2.10% (9)	+ 1.13% (17)
ST	+ 1.23% (3)	+ 3.26% * (26)
BFL	+ 1.15% (7)	+ 2.64% (22)
BFS	+ 0.12% (9)	0.00% (18)
Anterior-lateral leg	+ 0.57% (8)	+ 1.98% * (19)
TA	+ 0.87% (6)	+ 1.01% (23)
ELD	+ 0.75% (4)	+ 0.45% (25)
Pe	− 0.24% (3)	+ 2.24% (24)
Posterior leg	− 0.62% (5)	+ 1.82% ** (24)
So	+ 0.00% (4)	+ 1.43% * (26)
MG	+ 0.84% (7)	+ 1.84% (21)
LG	+ 0.24% (5)	+ 1.12% (25)

*P* value: * (< 0.05), **(< 0.005), ***(< 0.001). Statistically significant differences are reported in bold. In brackets the number of muscles in each group

### Correlations with clinical assessments

Correlations between clinical and quantitative MRI parameters at baseline are reported in Supplementary Table 1. The combined lower limb FF (thigh and leg) directly correlated with patient CSS (*r* = 0.65, *p* < 0.001) and inversely with 6MWT distance (*r* = − 0.69, *p* < 0.001). The combined lower limb wT2 had a significant correlation with CSS (*r* =  + 0.6, *p* < 0.005) and 6MWT (*r* = − 0.61, *p* < 0.001). The dynamometric assessment of knee extension and flexion negatively correlated with the quantitative MRI parameters (FF and wT2) of the anterior and posterior thigh compartments respectively, with the exception of the left anterior one. No significant correlation was found between FF or wT2 and EcoRI fragment length.

## Discussion

The research landscape on the use of MRI biomarkers in FSHD is evolving, with a few longitudinal studies assessing FF changes [14–17]. However, a recent dedicated workshop highlighted the potential of water mobility measures, such as wT2, as parameters affected early by the mechanisms of action of some investigational drugs. More data are needed to define the range of annual changes in MRI metrics for this heterogeneous disease, and a structured meta-analysis of individual data points has been proposed as a solution [4].

In this context, we explored the longitudinal changes in FF and wT2 in a cohort of FSHD subjects experiencing an “active” disease phase, characterized by at least one STIR positive muscle at baseline. In this population, we documented an average annual FF progression of approximately 2% in both the thigh and leg regions. When analyzing individual compartments, the anterior thigh demonstrated the highest annual FF increase (+ 3%), whereas other compartments exhibited increases below 2%. These findings align with previous reports from different FSHD populations, where mean annual FF changes ranged between 1.0% and 6.7% [14–17, 19].

Since we selected patients with fast-progressing disease, this value might appear lower than expected. However, they are higher than previously reported findings, such as a median 5-year change of 3.1% in a composite FF score for patients with ≥ 1 STIR positive muscle at baseline [18].

As FSHD progresses with a “muscle-by-muscle” fashion [31], we examined changes in individual muscles and observed substantial heterogeneity. Annual FF increases ranged from + 5.1% for RF and + 2.8% for VI, while many other muscles showed no significant changes over the study period. Interestingly, the RF exhibited unique behavior within our cohort. It had the lowest baseline wT2 value and was the only muscle with significant FF progression in both the wT2 subgroups (< 41 ms and > 41 ms). RF involvement, previously described in large cross-sectional studies, has been reported in younger, less clinically severe patients, particularly males [12]. In our cohort, approximately 20% were young males, suggesting that RF degeneration may progress rapidly and independently of edematous changes in this subgroup.

The variability in our results may also reflect technical factors, including MRI acquisition protocols (in our case 6-point Dixon for FF and 17-echoes T2 relaxometry for wT2), ROI segmentation, and post-processing methods used. Notably, Riem et al. highlighted that analytical variability is greater for smaller muscles during segmentation and quantification [32]. To address this issue, some authors have advocated for focusing on compartments rather than individual muscles, at least in certain patient cohorts [33, 34]. Heskamp et al. [35] provided compartment-level longitudinal FF data for seven FSHD patients, and our study contributes additional reference values for this simplified yet reliable analytical approach. However, this approach has the potential disadvantage of underestimating changes: for example, in our dataset the medial compartment did not show a significant increase in FF, probably because of the grouping of preferentially affected muscles (adductors) and usually spared muscles (e.g. sartorius, gracilis).

We observed no significant year-to-year changes in wT2 values, either globally or at the compartment/muscle level (Table 2). This finding aligns with previous studies reporting minimal changes in STIR positive muscle counts over one year [13]. Our analysis, using a quantitative approach, seems to corroborate these findings. Theoretically, inhibitors of DUX4 expression or its transcriptional activity could reduce disease activity and therefore wT2 values before affecting FF [4]. More research is needed to assess the responsiveness of MRI water mobility metrics, like wT2, in tracking the effects of targeted therapies.

To maximize the detection of therapeutic effects in FSHD muscles, selecting subgroups at higher risk of progression may be advantageous. We found that the most significant annualized FF changes occurred in muscles with either baseline intermediate FF levels (15–30%) or wT2 > 41 ms. This was consistent across individual muscles and compartments. Some muscles showed remarkable yearly FF changes also when moderately fatty-replaced. Conversely, compartments with wT2 < 41 ms showed no significant FF changes. Interestingly, in the anterior thigh, the subgroup with baseline FF > 30% demonstrated significant annualized FF increases (+ 5.8%, *p* < 0.001). This compartment could thus be a suitable target for longitudinal multiparametric MRI monitoring.

Previous studies have similarly reported higher FF progression in muscles with intermediate baseline FF levels. For example, Wang et al. found that muscles with FF between 40 and 50% showed the greatest FF increases, while those with FF below 10% or above 80% were least likely to progress over one year [16]. However, baseline FF cutoffs vary across studies, underscoring the need for harmonized datasets to better define threshold values and improve prognostic accuracy.

Moreover, as highlighted in a recent review, differences in longitudinal FF variations are influenced by factors such as methodological approaches, baseline FF levels, and the location and number of segmented slices, among others [36].

An increase in water content, detected via STIR or quantitative sequences, has been linked to higher FF progression risk [37]. In this study we applied wT2 as a quantitative parameter to detect edema and used a cutoff of 41 ms based on data from in-house healthy volunteers and literature reports [19, 30]. The average wT2 value for STIR negative muscles in our cohort (42 ms) exceeded this threshold, suggesting that STIR imaging may not detect low-grade inflammation. The transition from normal muscle to active disease likely occurs gradually, and linear quantification methods like wT2 may better capture this continuum at the cost of greater analytical complexity. Water T2 has not been widely used to monitor muscle edema in FHSD, with STIR imaging being the most common approach to detect increased water in muscle and to assess which muscles are most susceptible to fat replacement [15, 18, 21]. Technical difficulties have prevented so far a broader application of wT2 in quantitative MRI studies, including rather long acquisition times (especially if a wide body coverage is desired), the possible impact of elevated FF on wT2 determination, complex and demanding post-processing workflows [36]. Nevertheless, Dahlqvist et al. applied wT2 in their study of 10 FSHD subjects and already found that wT2 value increase fluctuated over time but ultimately persisted until complete fatty replacement occurred, and that higher values were always followed by more rapid fatty replacement [20].

Quantitative MRI-derived FF measurements correlate strongly with clinical scores and functional tests in FSHD [21] and other disorders [5]. Consistent with prior findings, we observed direct correlations between FF and CSS, and inverse correlations between FF and both 6MWT and dynamometric assessments. These results are expected, as higher FF corresponds to weaker muscles, poorer functional performance, and greater disability. More intriguingly, similar correlations were found between clinical measures and wT2 values, suggesting that wT2 is linked to muscle performance in FSHD. This relationship has also been documented in other neuromuscular diseases such as inclusion body myositis [38], hereditary transthyretin amyloidosis [39], and amyotrophic lateral sclerosis [40]. While denervation explains these findings in some disorders, inflammatory changes alongside atrophy are likely the primary source of elevated wT2 in FSHD and IBM.

We acknowledge that the lack of a control group is a limitation of our study design, which was focused on defining the radiological natural history of a specific cohort of FSHD patients rather than comparing affected vs unaffected individuals. We thus cannot draw definite conclusions whether or not the differences observed in our population lie within the variations expected in healthy volunteers. As a reference, repeatability quantitative MRI studies available in literature assessing FF stability over time esteemed a coefficient of variation ranging from 1.6 to 6.3% on average [41, 42]. The small cohort size is another limitation of our study. Nevertheless, the extended follow-up period (24 months) provided valuable insights into disease trajectories. Multiple time points enabled the use of robust mixed models, enhancing reliability. Furthermore, since we selected a specific group of patients with a high number of STIR positive muscles, our results may not be generalizable to the entire FSHD population. A larger sample size would further strengthen statistical power, allowing the integration of additional baseline clinical and radiological parameters for comprehensive risk stratification.

## Conclusions

In conclusion, our 2-year longitudinal MRI study of lower-limb muscles in FSHD identified muscles and compartments at higher risk of disease progression. These findings can guide clinical trial design and the selection of outcome measures. Multiparametric quantitative MRI metrics should be incorporated into clinical trial frameworks to explore their potential in detecting early therapeutic effects.

## Supplementary Information

Below is the link to the electronic supplementary material.

Supplementary file1 (DOCX 1100 KB)

Supplementary file2 (DOCX 16 KB)

## Data Availability

The original contributions presented in the study are publicly available. Raw MRI data are available in the Zenodo repository: 10.5281/zenodo.14216594.
