# Supplementary material for: Natural history of facioscapulohumeral muscular dystrophy evaluated by multiparametric quantitative MRI: a prospective cohort study
Source: J Neurol. 2025 Apr 2;272(4):306. doi: 10.1007/s00415-025-13062-8 (PMC11965262; doi:10.1007/s00415-025-13062-8)

Supplementary Fidures: per-muscle yearly fat-fraction change in relation to the baseline fat-fraction (expressed in percentile bins).


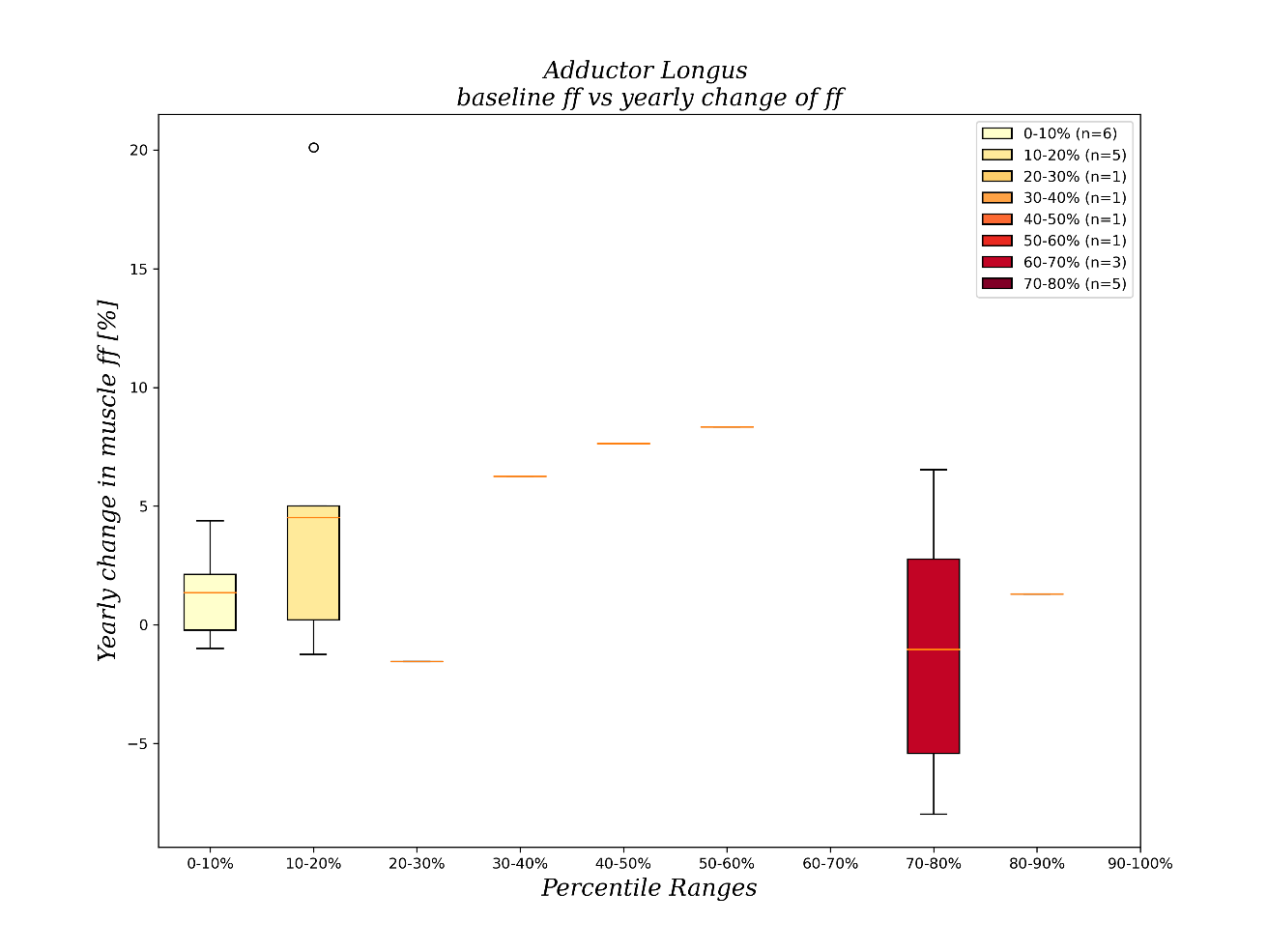


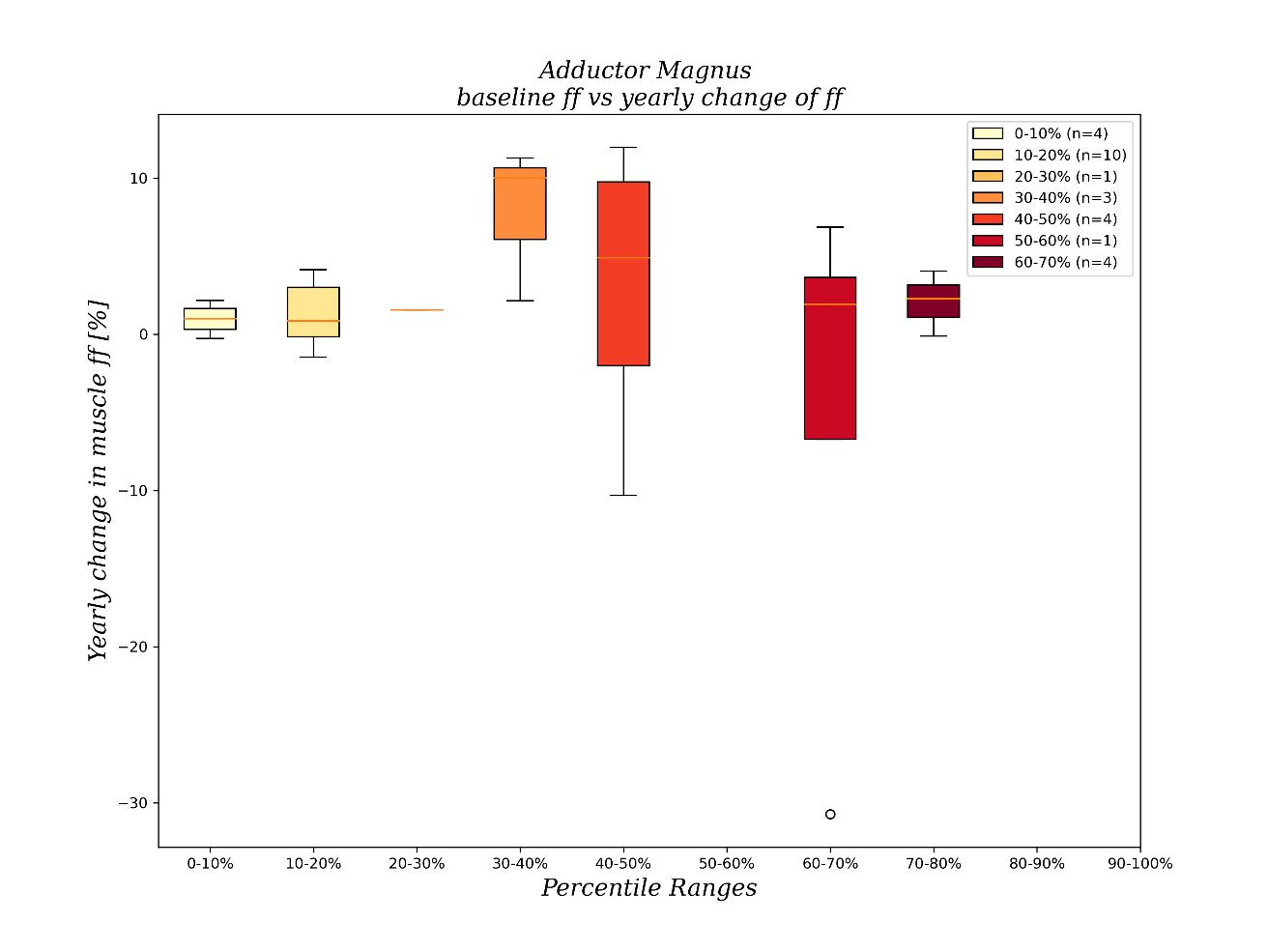


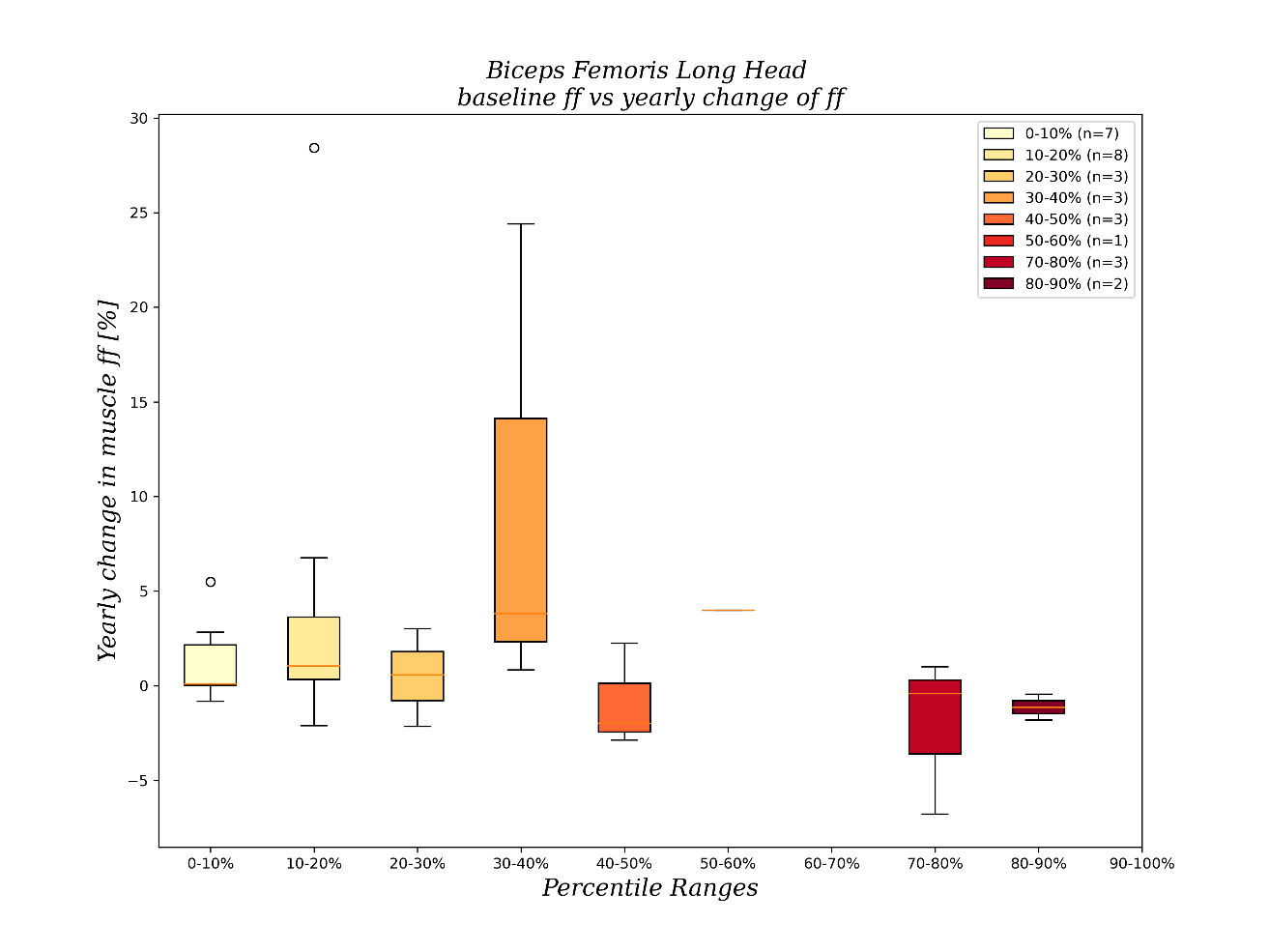

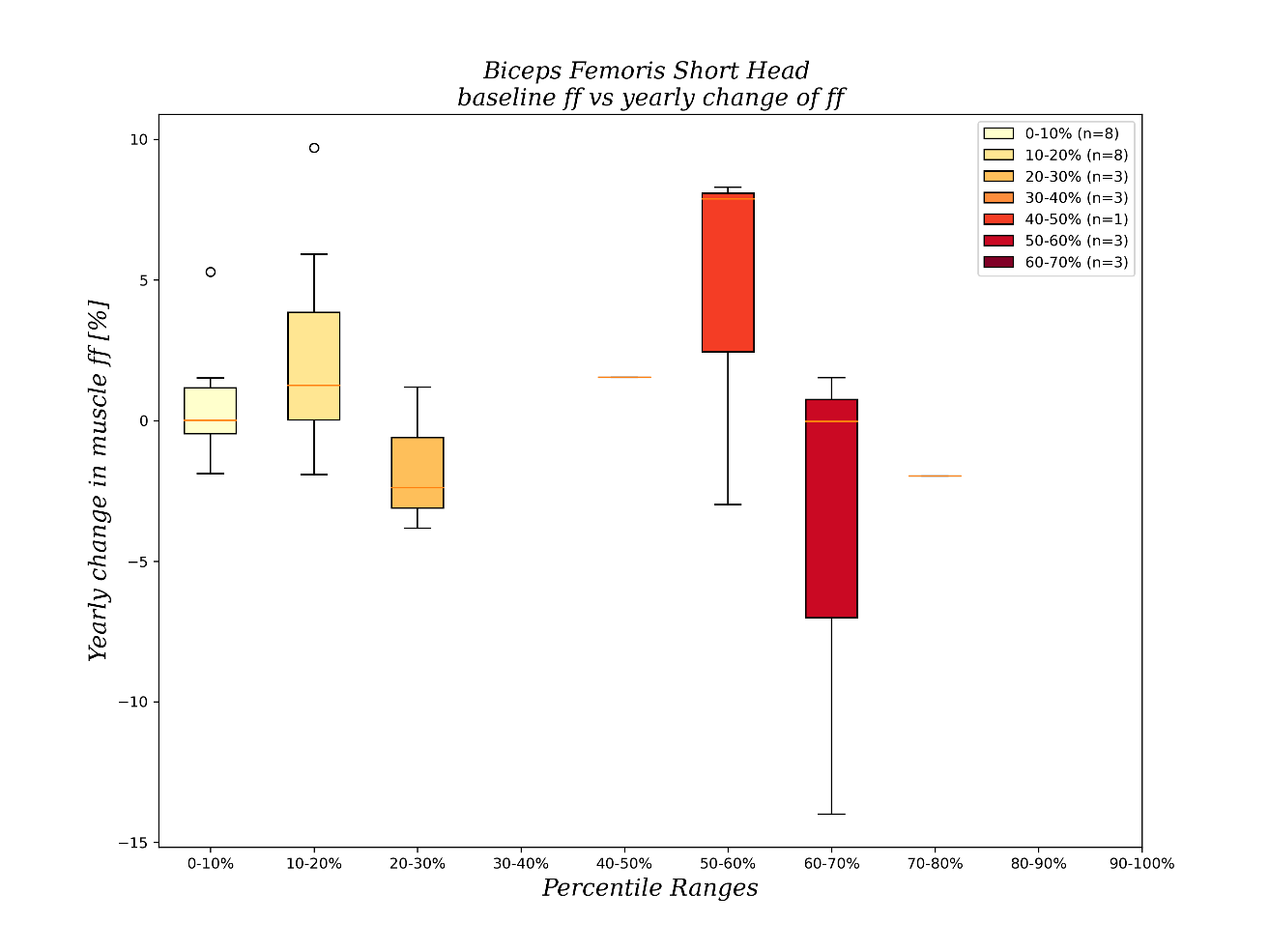


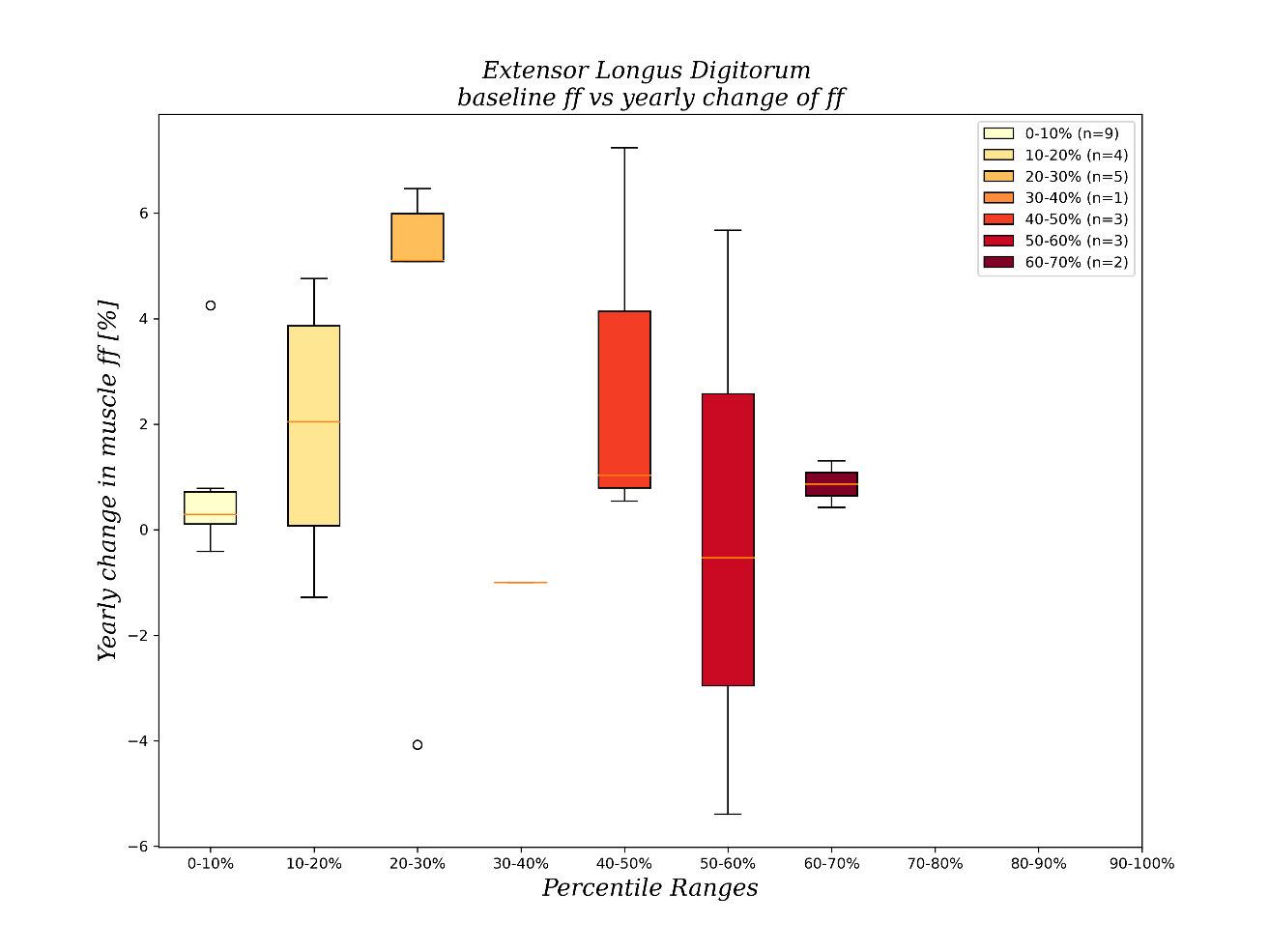

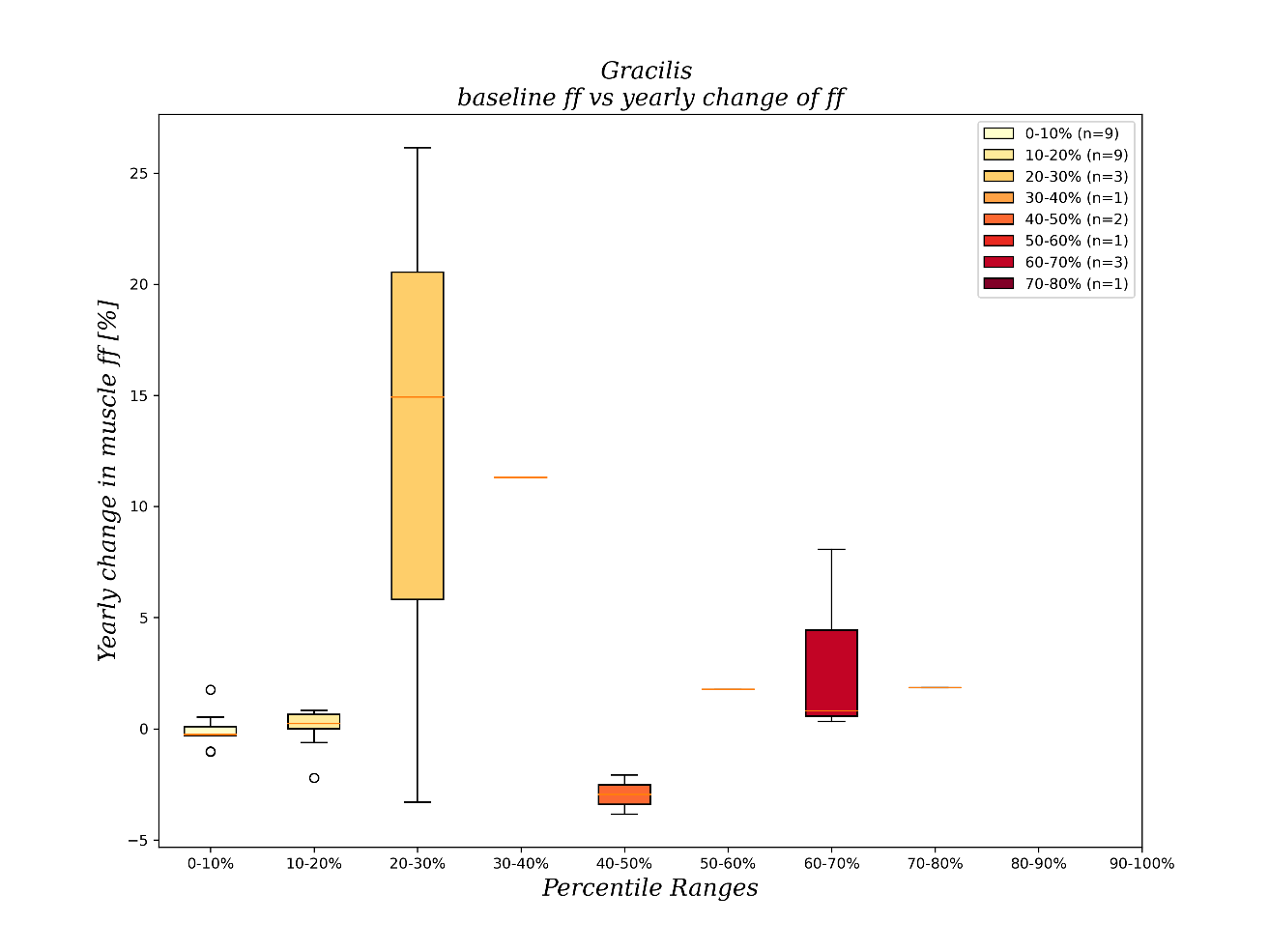


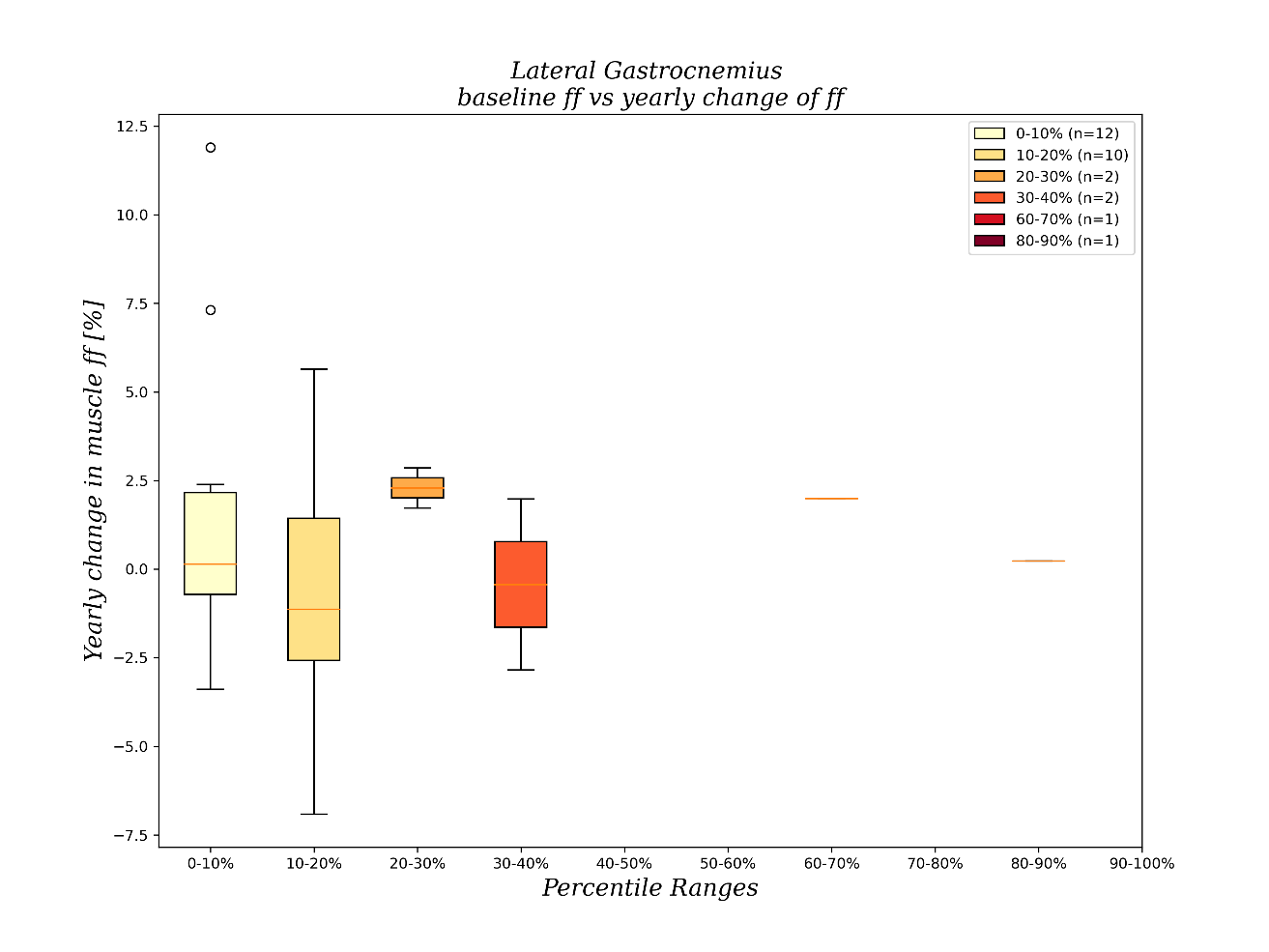

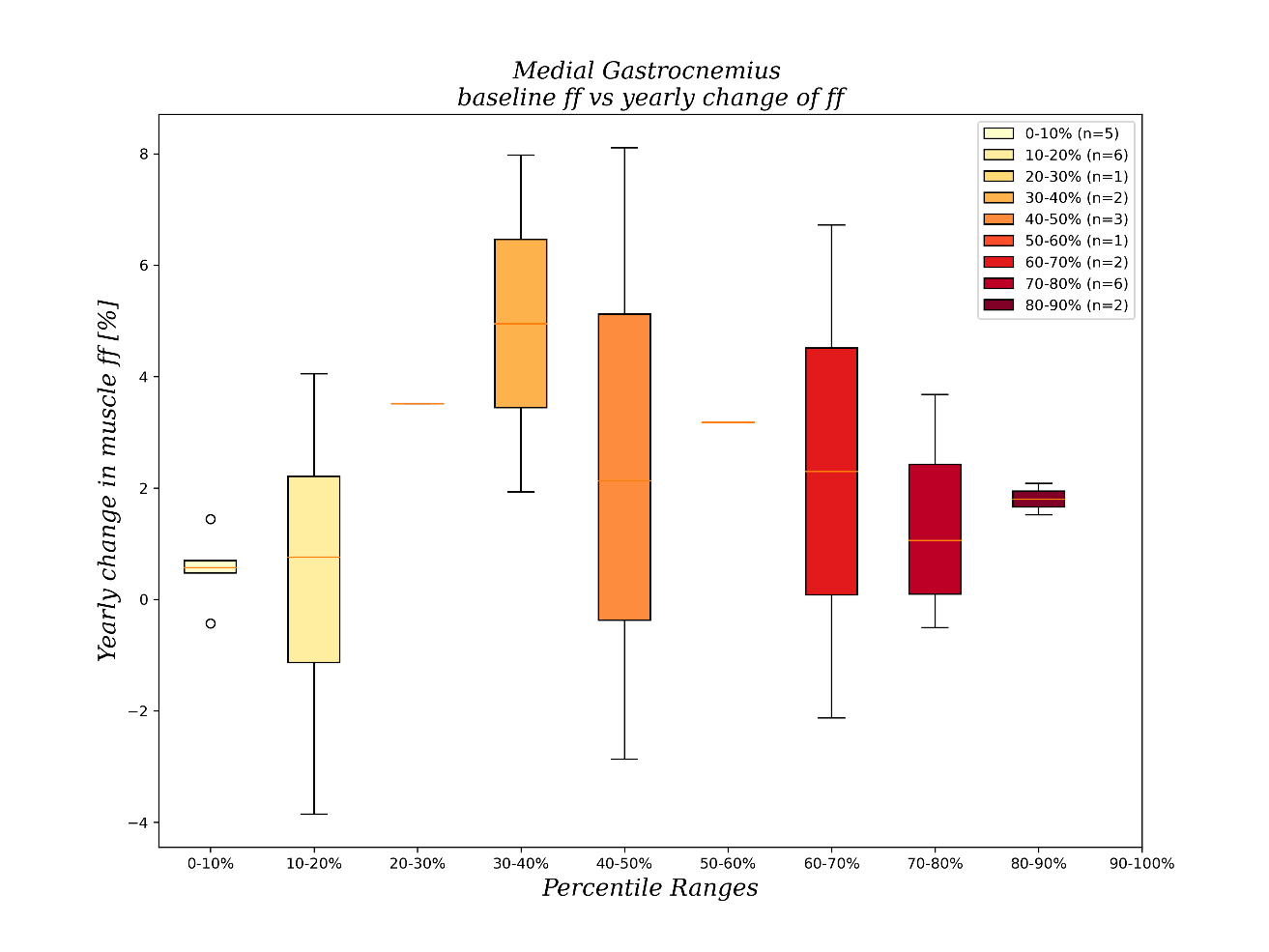


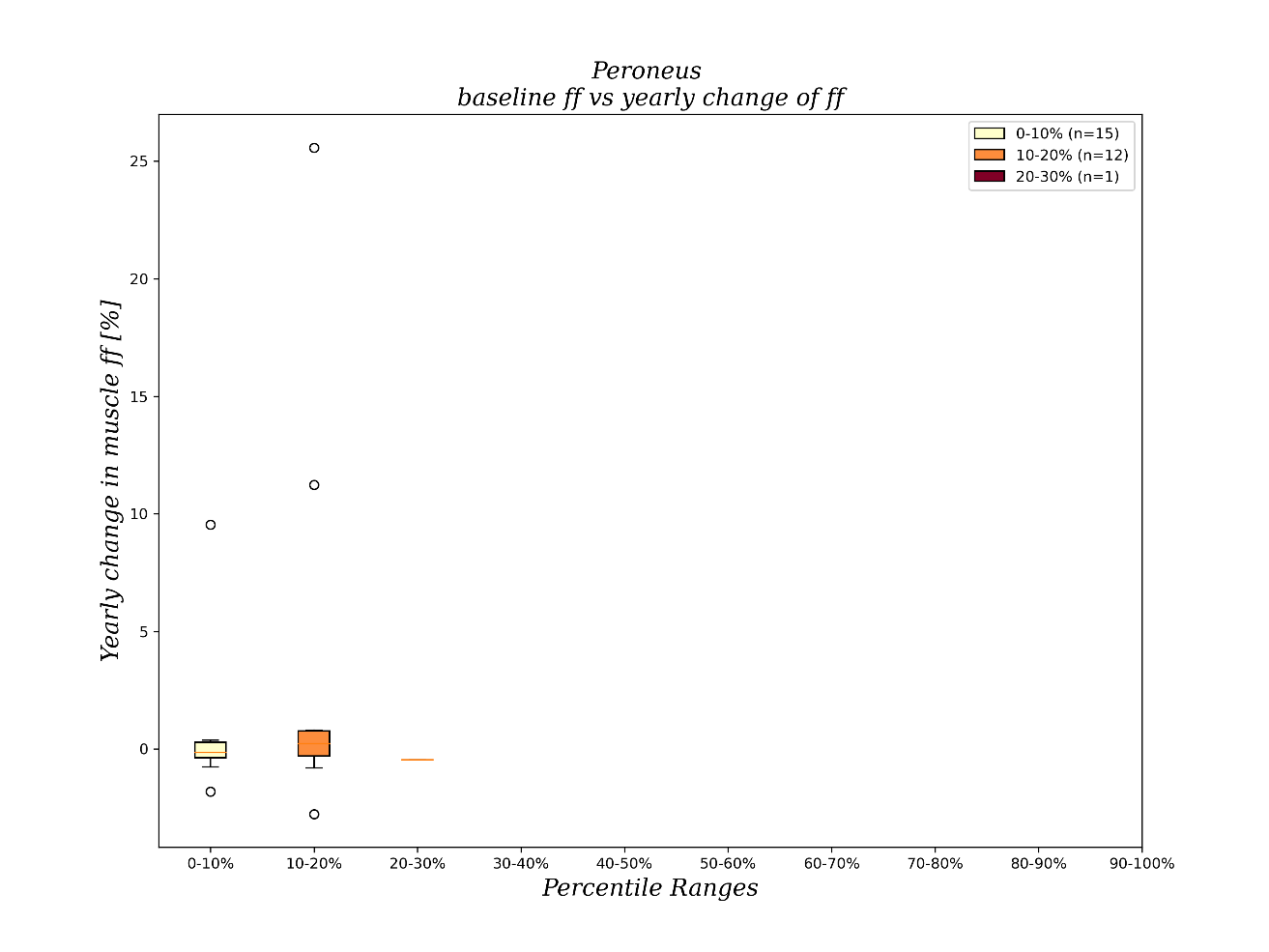

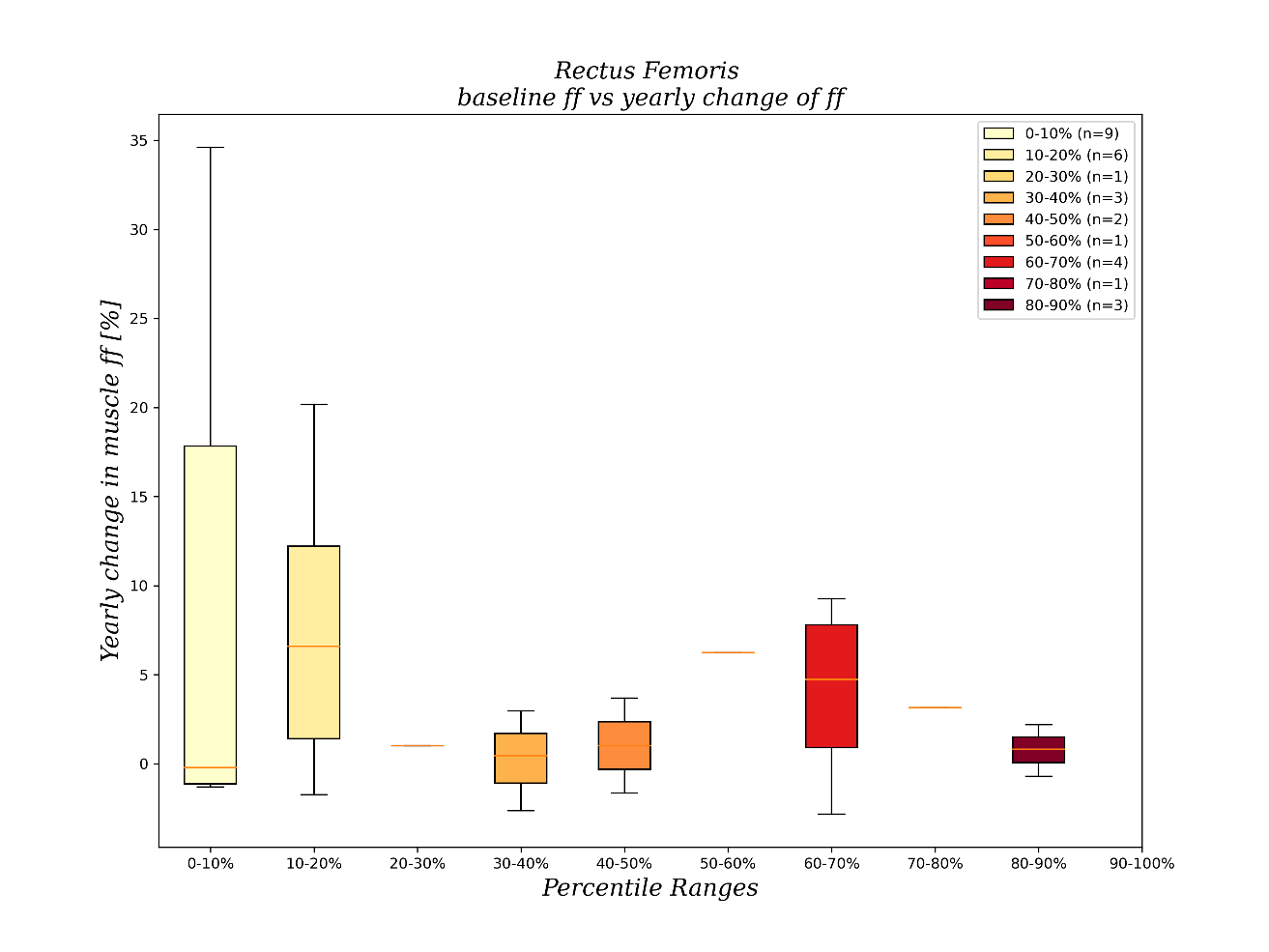


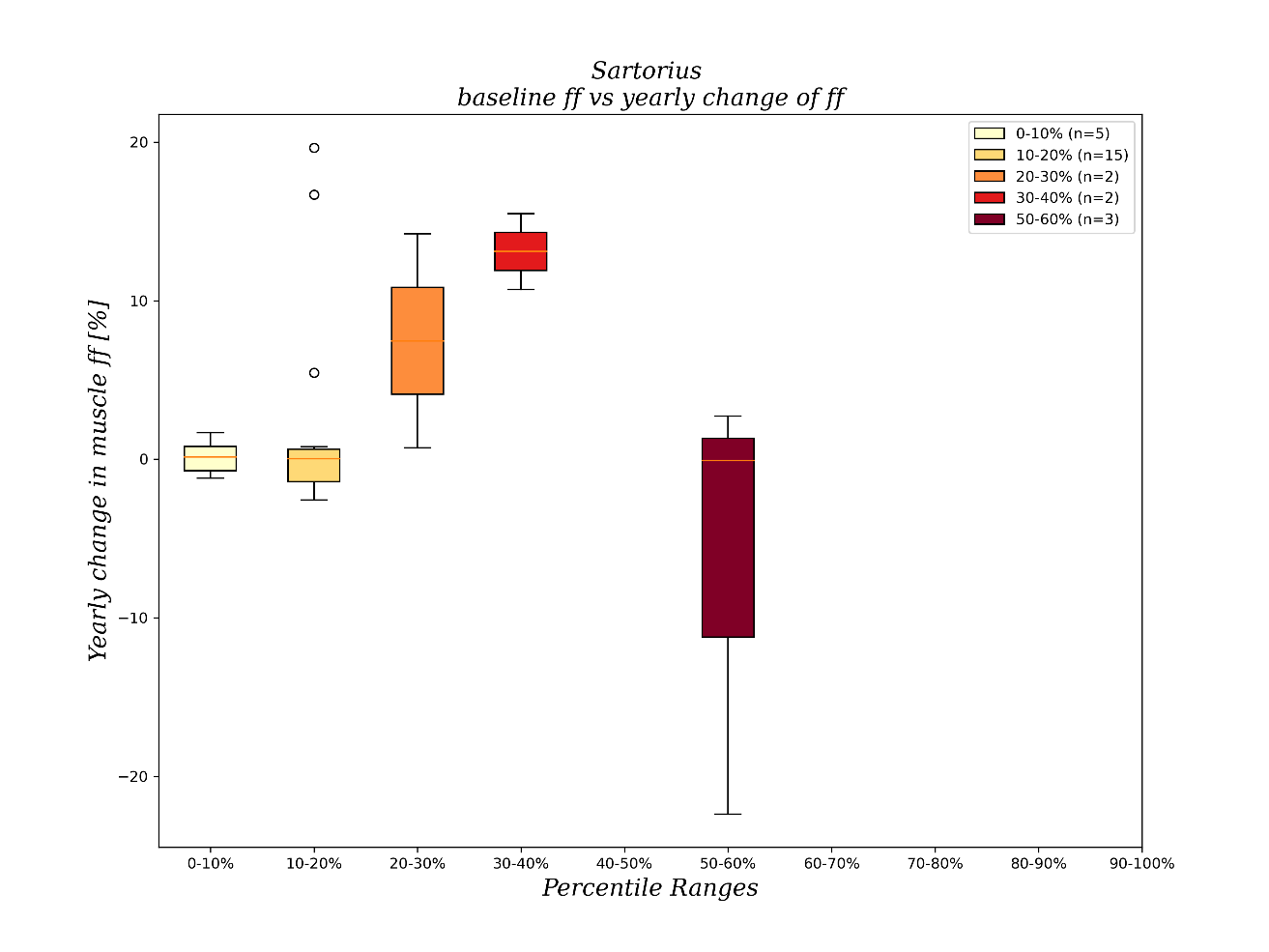

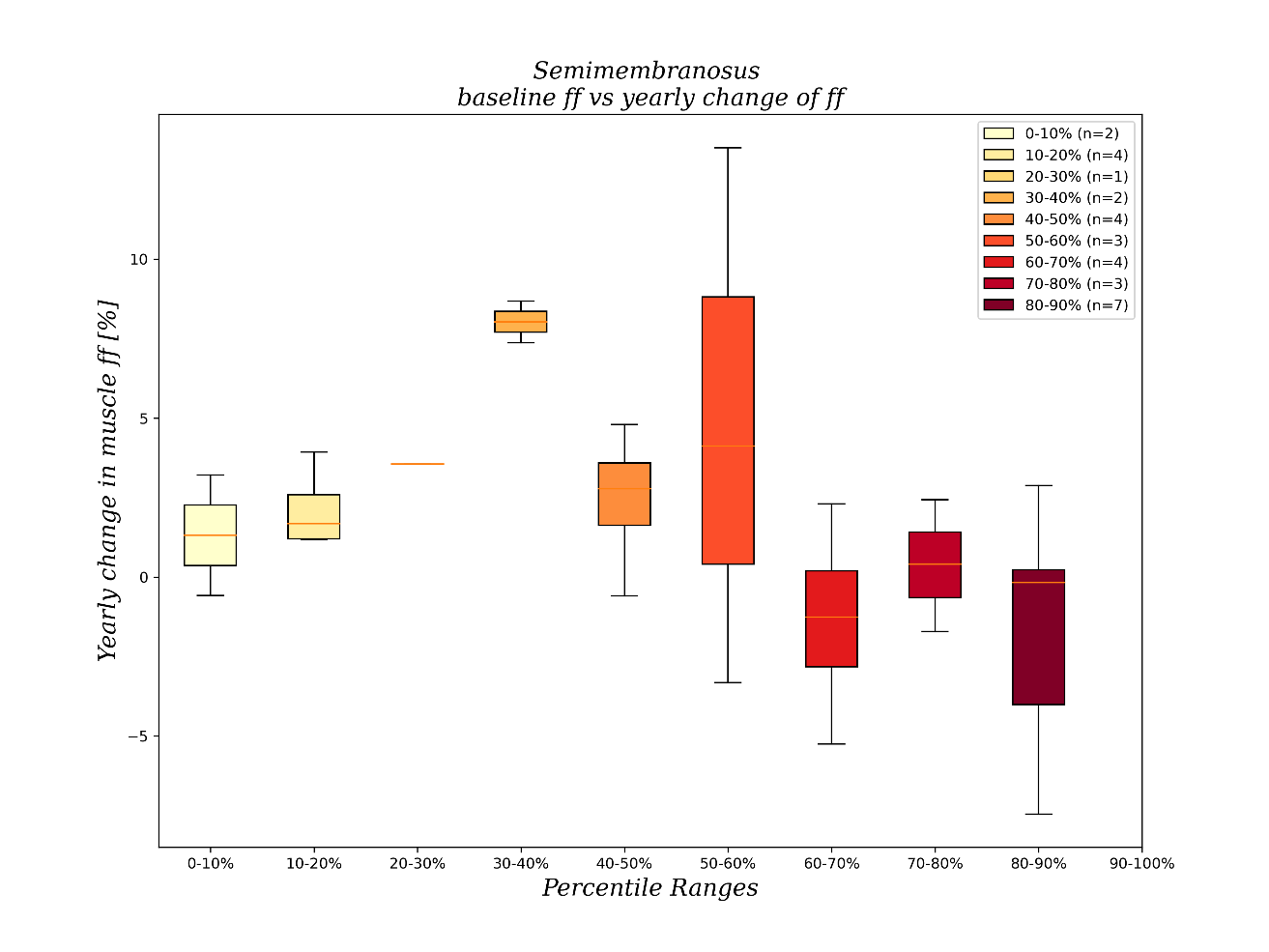


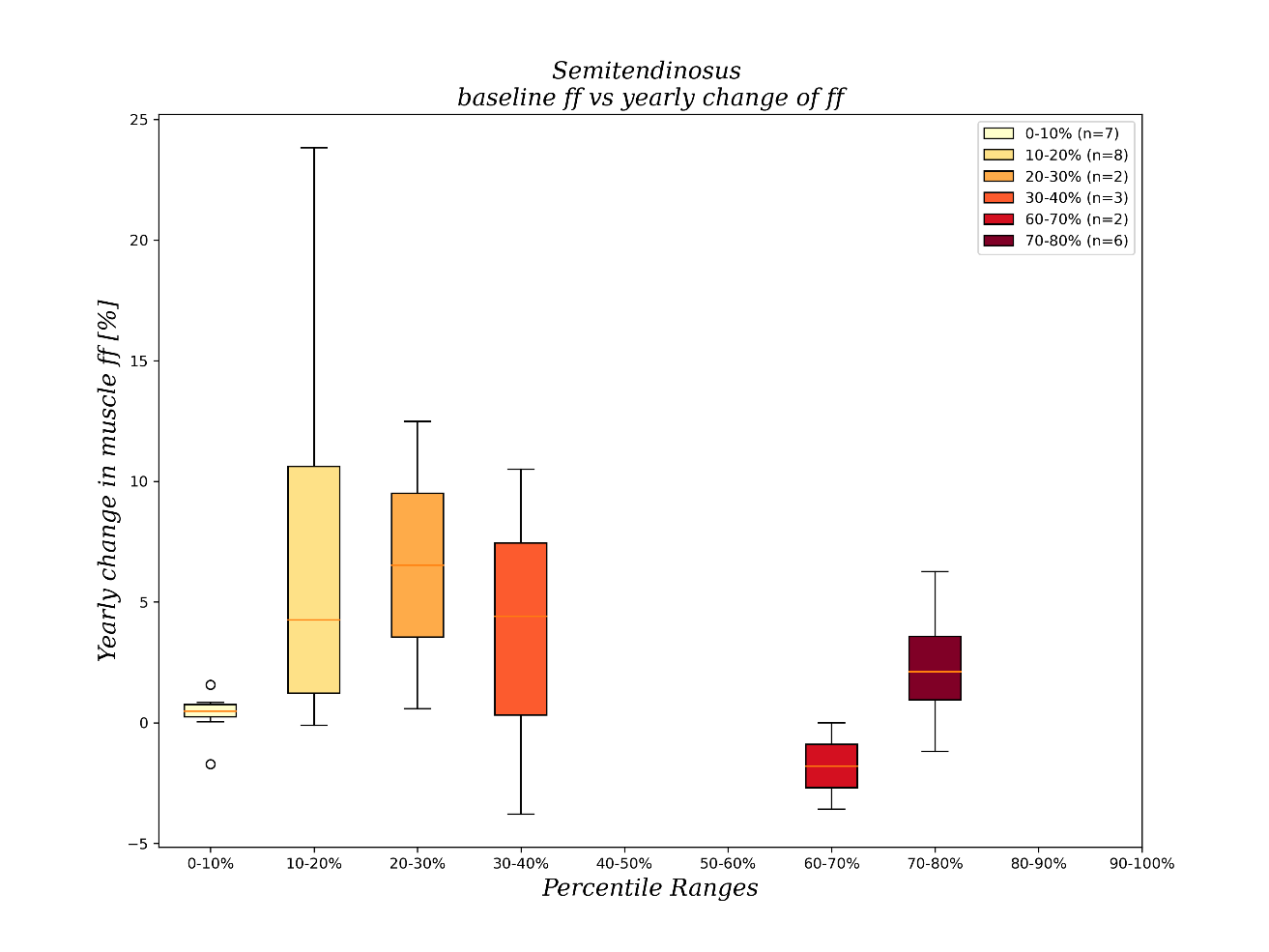

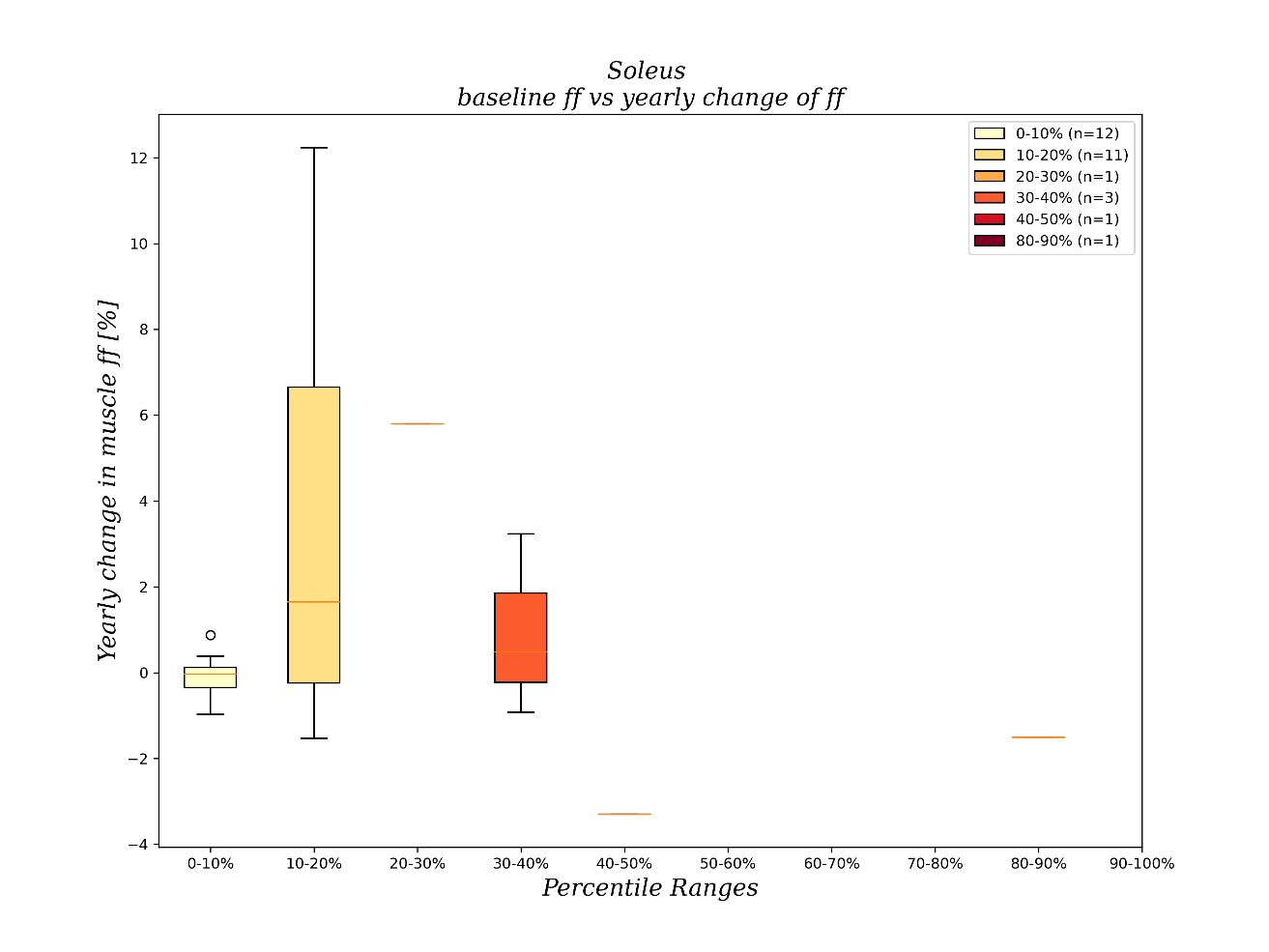


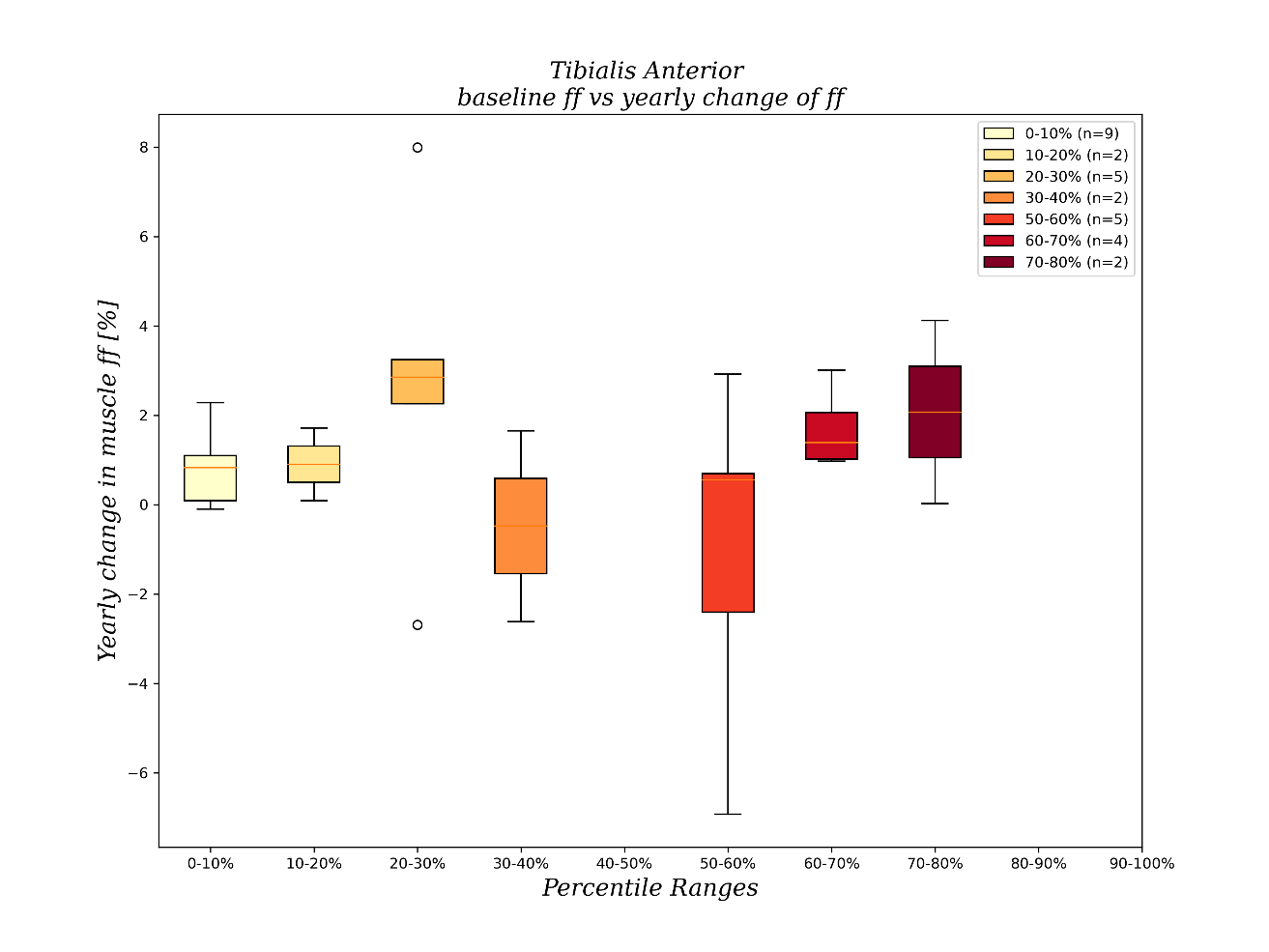

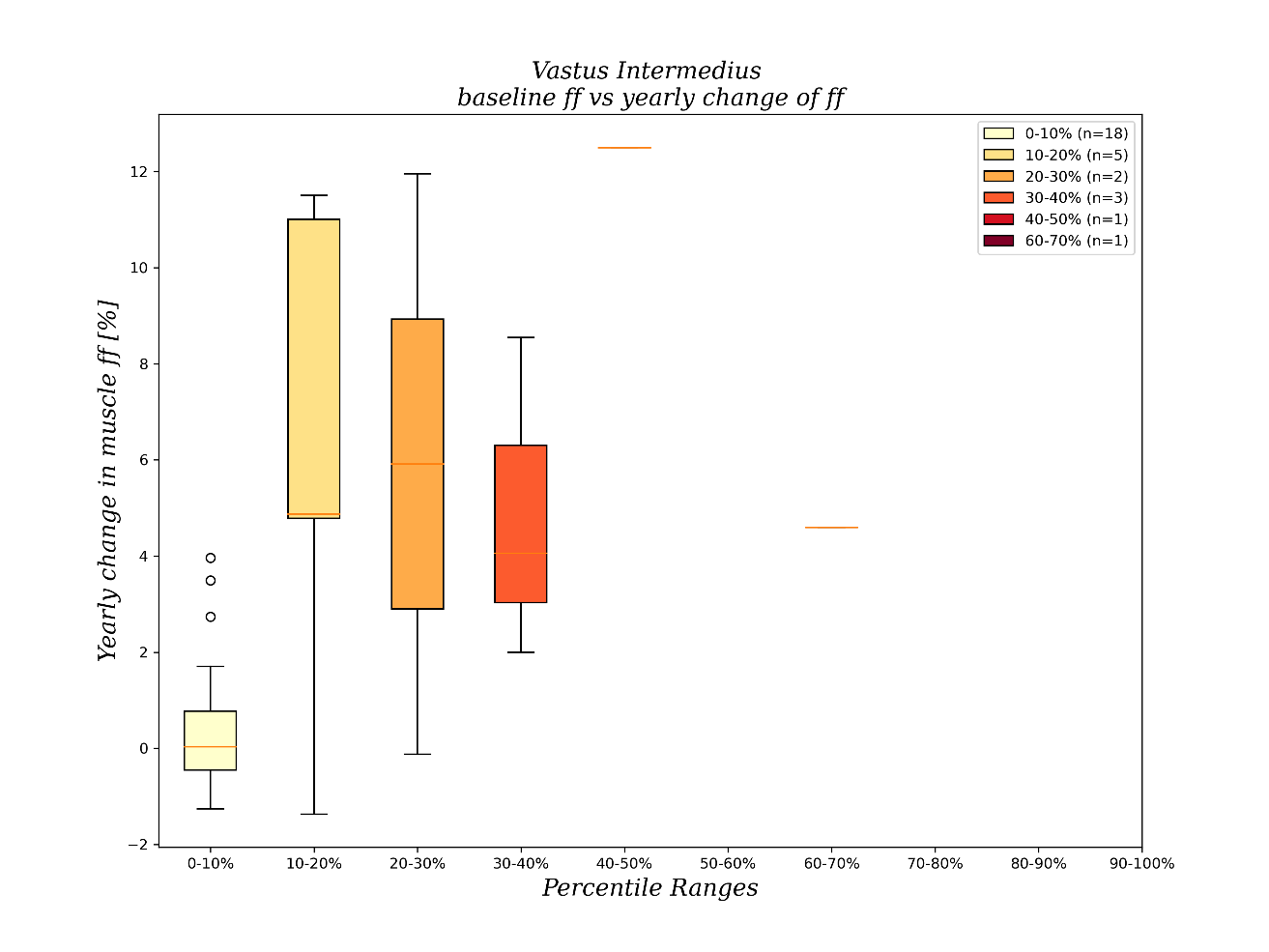


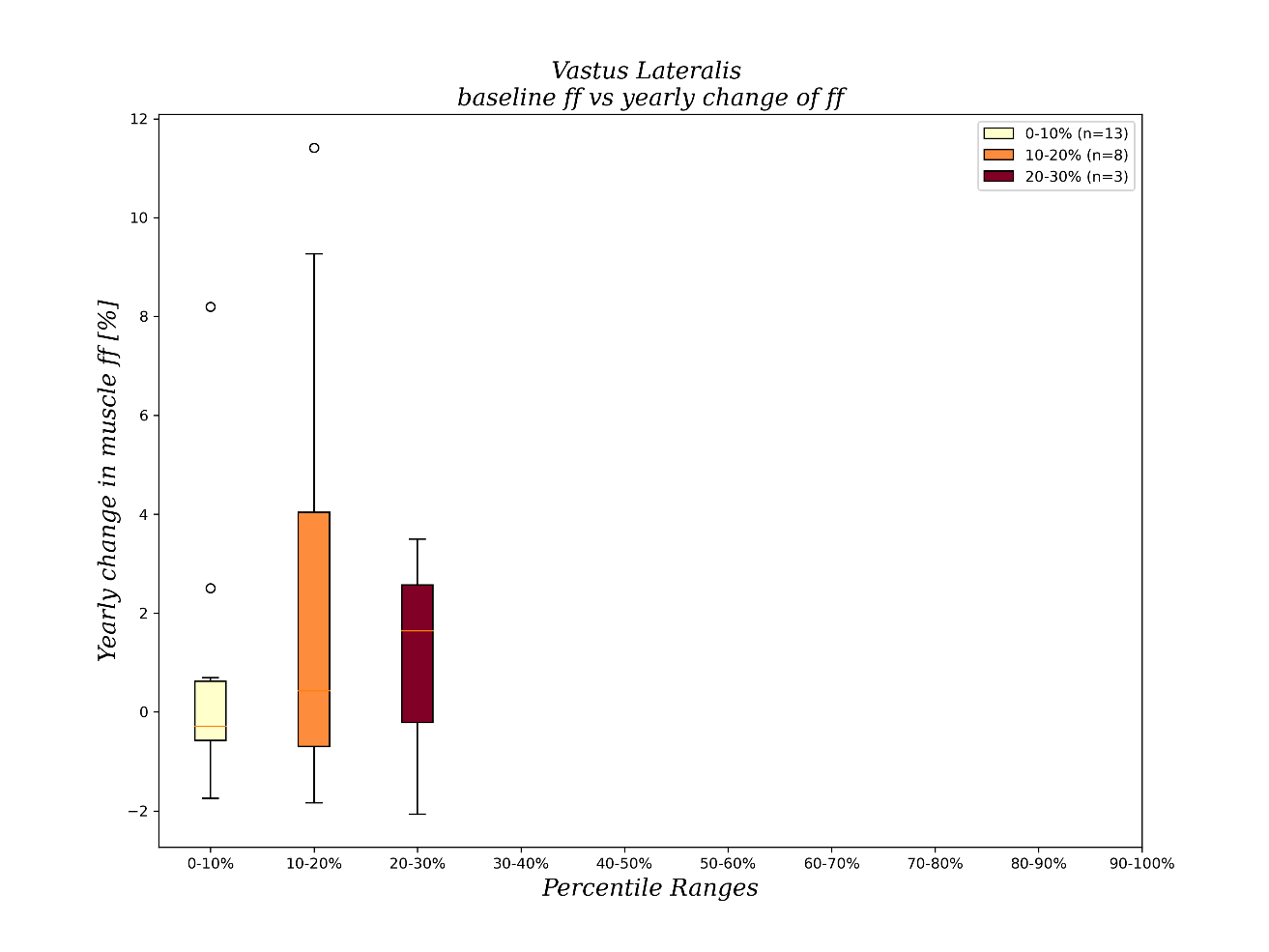

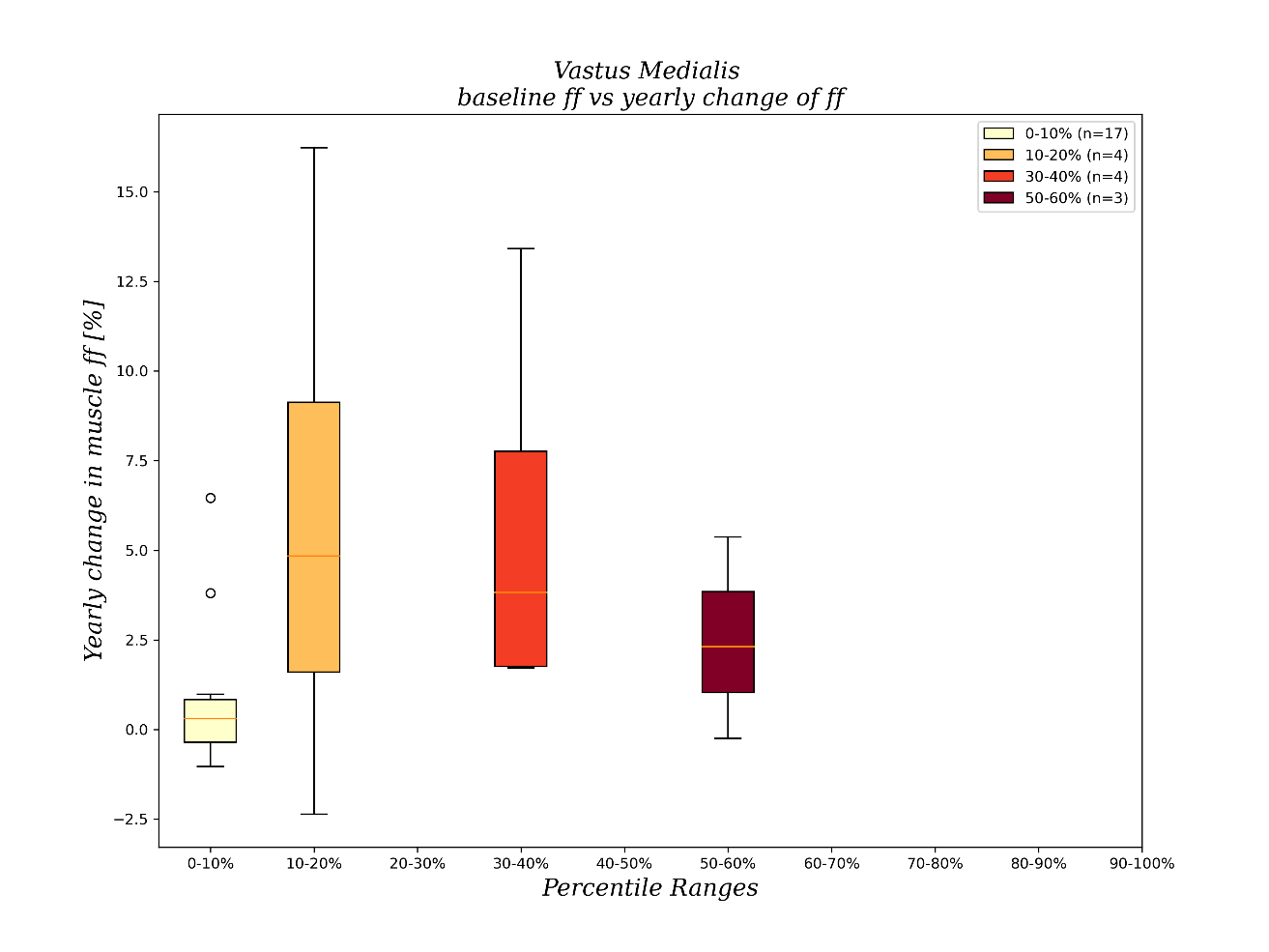

Supplement: Supplementary file 1 — Supplementary file1 (DOCX 1100 KB) [file 415_2025_13062_MOESM1_ESM.docx]
