# Supplementary material for: Natural history of facioscapulohumeral muscular dystrophy evaluated by multiparametric quantitative MRI: a prospective cohort study
Source: J Neurol. 2025 Apr 2;272(4):306. doi: 10.1007/s00415-025-13062-8 (PMC11965262; doi:10.1007/s00415-025-13062-8)
Supplement: Supplementary file 2 — Supplementary file2 (DOCX 16 KB) [file 415_2025_13062_MOESM2_ESM.docx]

**Supplementary Table 1.** Pearson’s correlations (rho) between clinical tests and FF and wT2 at baseline, 12 months and 24 months follow-up. CSS and 6-MWT were correlated with lower limb FF and wT2 (combining thighs and legs); dynamometry of anterior and posterior thigh (Dyn knee ext L, Dyn knee ext R, Dyn knee flex L, Dyn knee flex R) compartments were correlated with corresponding compartments FF and wT2. p value: * (<0.05), **(< 0.005), ***(<0.001).

| **Region** | **MRI parameter** | **Clinical test** | **Correlation coefficient** |
| --- | --- | --- | --- |
| Lower limb | FF | CSS | **+0.65 ***** |
|  | wT2 | CSS | **+0.6 **** |
|  | FF | 6-MWT | **-0.69 ***** |
|  | wT2 | 6-MWT | **-0.61***** |
| Anterior Thigh | FF left | Dyn knee ext L | -0.28 |
|  | FF right | Dyn knee ext R | **-0.52 **** |
|  | wT2 left | Dyn knee ext L | -0.27 |
|  | wT2 right | Dyn knee ext R | **-0.51 *** |
| Posterior Thigh | FF left | Dyn knee flex L | **-0.76 ***** |
|  | FF right | Dyn knee flex R | **-0.8 ***** |
|  | wT2 left | Dyn knee flex L | **-0.58 **** |
|  | wT2 right | Dyn knee flex R | **-**0.11 |
